# Supplementary material for: Developmental genomics of limb malformations: Allelic series in association with gene dosage effects contribute to the clinical variability
Source: HGG Adv. 2022 Aug 4;3(4):100132. doi: 10.1016/j.xhgg.2022.100132 (PMC9403727; doi:10.1016/j.xhgg.2022.100132)
Supplement: Document S1. Figures S1–S7 [file mmc1.pdf]

Supplemental information

Developmental genomics of limb malformations:

Allelic series in association with gene

dosage effects contribute to the clinical variability

Ruizhi Duan, Hadia Hijazi, Elif Yilmaz Gulec, Hatice Koçak Eker, Silvia R. Costa, Yavuz Sahin, Zeynep Ocak, Sedat Isikay, Ozge Ozalp, Sevcin Bozdogan, Huseyin Aslan, Nursel Elcioglu, Débora R. Bertola, Alper Gezdirici, Haowei Du, Jawid M. Fatih, Christopher M. Grochowski, Gulsen Akay, Baylor-Hopkins Center for Mendelian Genomics, Shalini N. Jhangiani, Ender Karaca, Shen Gu, Zeynep Coban-Akdemir, Jennifer E. Posey, Yavuz Bayram, V. Reid Sutton, Claudia M.B. Carvalho, Davut Pehlivan, Richard A. Gibbs, and James R. Lupski

Table of Contents

**SUPPLEMENTAL NOTE: CASE REPORTS**	**2**
BAB4812	2
BAB6263	3
BAB11205	3
**SUPPLEMENTAL FIGURES**	**5**
Figure S1	5
Figure S2.....	7
Figure S3.....	8
Figure S4.....	10
Figure S5.....	13
Figure S6.....	15
Figure S7.....	16
**SUPPLEMENTAL MATERIAL AND METHODS**	**17**
PCR Primers used in this study	17

SUPPLEMENTAL NOTE: CASE REPORTS

BAB4812

BAB4812 is a male, born via C-section at 34 weeks of gestation per ultrasound measurements (38 4/7 per last menstrual period), with a birth weight of 1.88 kg (10-25%ile per 34 GW measurements). His parents were from the same Turkish village with no report of consanguinity. The index subject has a brother who was affected by hydrocephalus and died at 7-months of age, and the parents later had a healthy girl from their second pregnancy. The index subject was their third child. By physical examination at seven days of life, the subject weighed 2 kg (3-10%ile per 35 GW measurements) and measured 31.5 cm (10-50%ile per 35 GW measurements) in length. He had hypotonia, down-slanting palpebral fissures, micrognathia, *pectus carinatum*, bilateral split-hand malformation, bilateral syndactyly involving the 3rd and 4th toes, and ‘paleness’ on the inner sides of the legs (**Figure S4**). Laryngomalacia and unilateral vocal cord paralysis were identified by the ear nose & throat examination. The echocardiogram on the proband showed systolic dysfunction and valve insufficiency, and a troponin I was mildly elevated at 0.07 ng/ml (normal < 0.04 ng/ml) and attributed to perinatal asphyxia. Ophthalmologic evaluation was remarkable for bilateral intraretinal hemorrhages and lack of vascularization in the periphery of zone 3. Karyotype was normal, 46,XY. During the evaluation at 2-months of age, the index subject was hypotonic with a poor suck reflex. Anthropometric measurements revealed 2.8 kg (3%ile) weight and 34 cm (-7.3 *SD*) length. The proband was diagnosed with intellectual disability at 7 years old with delays in all developmental parameters. The most recent clinical evaluation at 8.5-years revealed measurements of 15 kg (-3.7 *SD*) weight and 102 cm (-4.9 *SD*) height. He was unable to speak or swallow, and had vomiting

(likely secondary to reflux). He had no appetite, could not eat solid food and was fed with formula. He had constipation without alternating diarrhea episodes. Laboratory tests including complete blood count, liver function tests, and thyroid function tests yielded unremarkable values. The celiac disease antibody test was negative.

BAB6263

BAB6263 was an 11-year-old male who developed seizures at 3-months of age. His parents were 1st cousins from Turkey. The subject displayed intellectual disability (ID) during the latest examination, and his brain MRI showed mild cerebellar atrophy (**Figure S3**). His renal ultrasound was normal. No additional abnormalities were identified in limb radiographs. He has two healthy sisters and two brothers (BAB6262 and BAB8610) who were only affected with split-hand/foot malformation and had normal cognition.

BAB11205

BAB11205 was a male subject and the twin product of *in vitro* fertilization. His twin resulted in miscarriage during pregnancy (**Figure S7**). His parents were from nearby villages with no report of known consanguinity. His parents reported three previous miscarriages. Prenatal ultrasound revealed short long bones and an increased nuchal fold. At 7-months of age, physical examination showed his head circumference to be 39 cm (-4 *SD*), weight 4.75 kg (-4.2 *SD*), and length 58 cm (-4.3 *SD*). At the 16-months of age evaluation, anthropometric measurements were: head circumference 42 cm (-4.1 *SD*), weight 7.4 kg (-3.5 *SD*) and height 70 cm (-3.3 *SD*). Physical examination was remarkable for microcephaly, open anterior fontanelle, narrow forehead, blepharophimosis, ptosis, thin lips, long fingers with partial syndactyly of left hand, bilateral single transverse palmar crease, short and bowed femurs, bilateral cryptorchidism, and

bilateral oligodactyly of the feet (**Figure S7**). Abdominal ultrasound showed left renal agenesis. Echocardiogram revealed multiple cardiac anomalies, including atrial septal defect, ventricular septal defect, peripheral pulmonary stenosis and mitral valve prolapse. No abnormal findings were noted on his electroencephalogram (EEG). His karyotype was normal 46, XY. Cranial MRI showed mild triventricular hydrocephalus. A 60K array CGH did not reveal any copy number variations.

SUPPLEMENTAL FIGURES

Figure S1

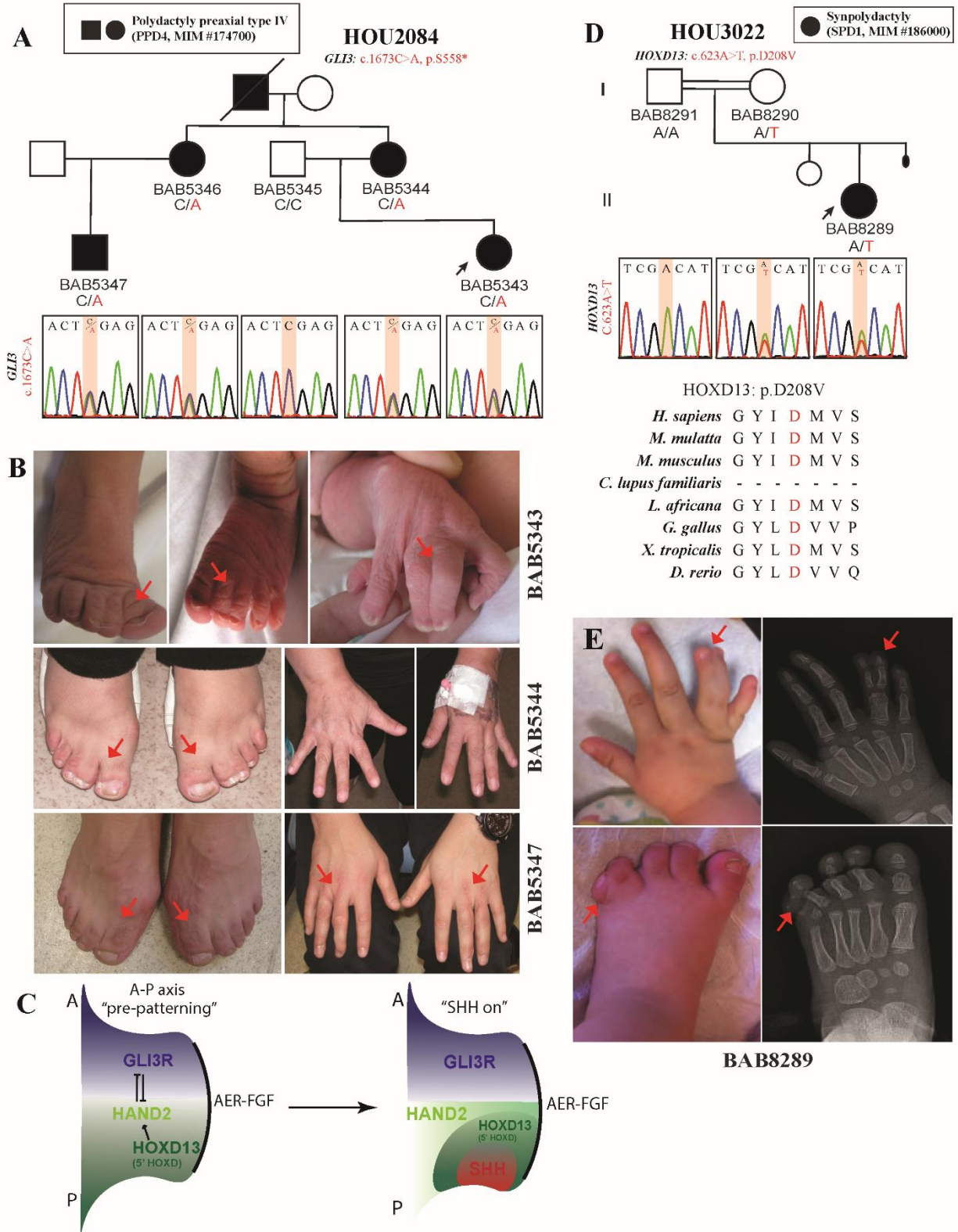

Figure S1. Novel variant identification involving genes contributing to A/P axis patterning

- A. A simplified pedigree of HOU2084 with selected family members. Novel nonsense variant (*GLI3*, c.1673C>A) profiled by four-color Sanger sequencing chromatogram, segregated with the PPD4 (MIM#174700) rare disease trait in accordance with Mendelian expectations for a dominant trait.
- B. Clinical photos show the dorsal views of hands and feet from three selected affected individuals. From top to bottom: proband (BAB5343), affected mother of the proband (BAB5344), and maternal cousin of the proband (BAB5347). Red arrows identify the common congenital limb malformation (CLM) features shared by the three affected individuals, including broad/duplicated halluces and 2-3 toe syndactyly and 3-4 finger syndactyly (note post-surgical scar in the hand of subject BAB5347)
- C. The left illustration describes the limb bud. Mutual genetic antagonism between the short isoform of *GLI3* (*GLI3R*) and *HAND2* is required to establish a “pre-patterning” of the A/P axis before forming the *SHH*-ZPA gradient. *HOXD13* expression is essential to sustain the *HAND2* activation. The right diagram shows that the counterregulatory interactions among *GLI3R*, *HAND2* and *HOXD13* continue to orchestrate limb bud A/P polarization after establishing the *SHH* gradient.
- D. Pedigree structure of HOU3022/BAB8289 and segregation shows the maternally inherited missense variant allele (*HOXD13*, c.623A>T). The altered residue (p.D208V) is evolutionarily conserved.
- E. Clinical photos and radiographs show the observed SPD1 phenotype of proband BAB8289. Photos show 4th finger polysyndactyly and 4-5 toe syndactyly; radiographs of the hand show duplication of the 4th finger phalanges and hypoplasia of the distal phalanx of the 5th finger; radiographs of the feet show a delta proximal 5th phalanx (longitudinally bracketed epiphysis) and hypoplastic distal phalanges of the great toe and 5th toe.

Figure S2

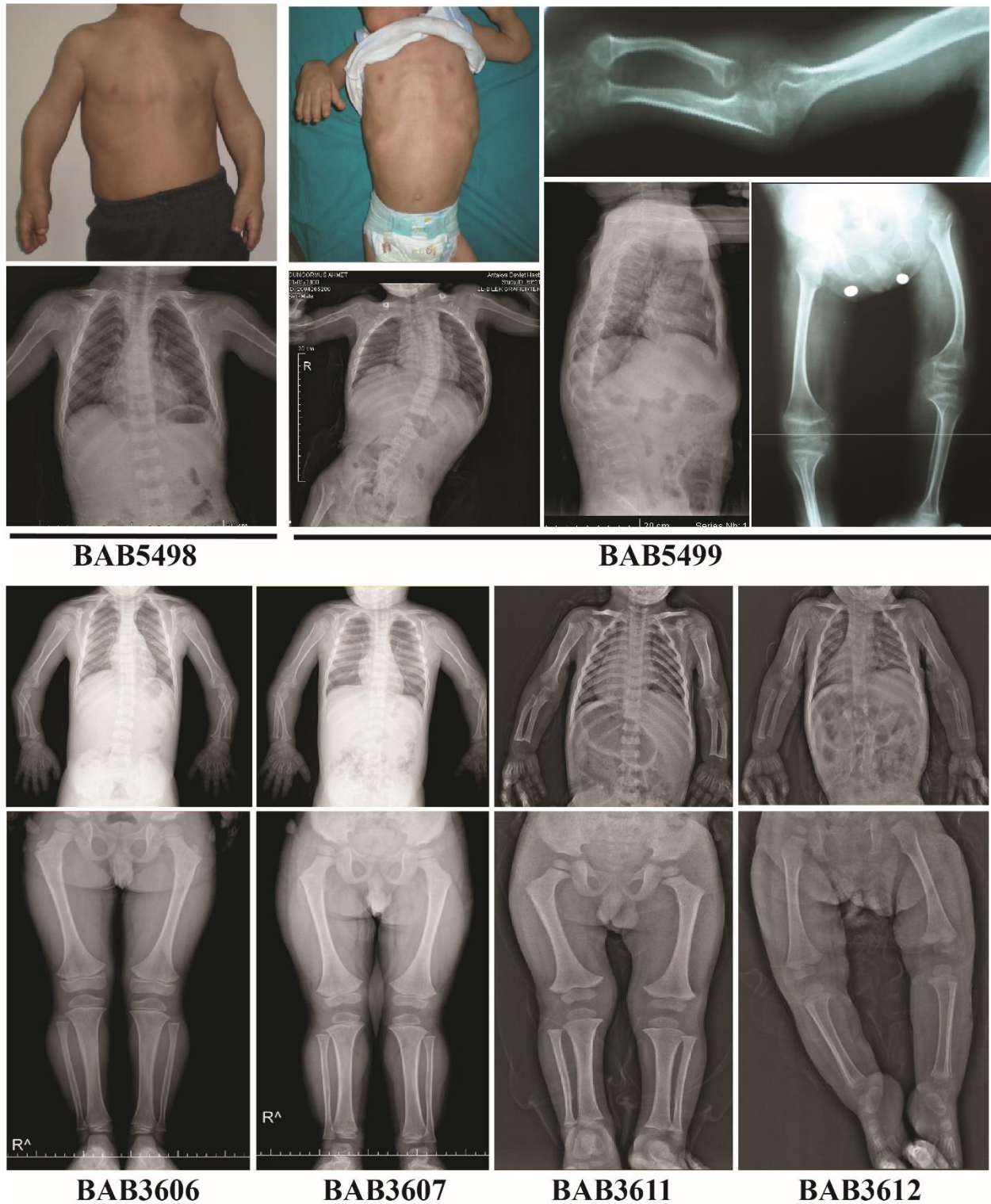

Figure S2. Clinical photographs and radiographs of six individuals with Acromesomelic dysplasia, Maroteaux type (AMDM) from three unrelated Turkish families. Upper panel:

BAB5498 and BAB5499 images demonstrate *pectus excavatum* and micromelia with predominant rhizomelia; radiographs show a short, bowed forearm, platyspondyly, thoracic kyphosis, and congenital hip dislocation in individual BAB5499. Bottom panel: Radiographs from four affected individuals show short upper limbs with short and thick humeri and even more pronounced shortening and bowing of the forearms, symmetrically short and broad metacarpals, and phalanges, varying degrees of platyspondyly, flattened acetabulum and *coxa vara* in individual BAB3612.

Figure S3

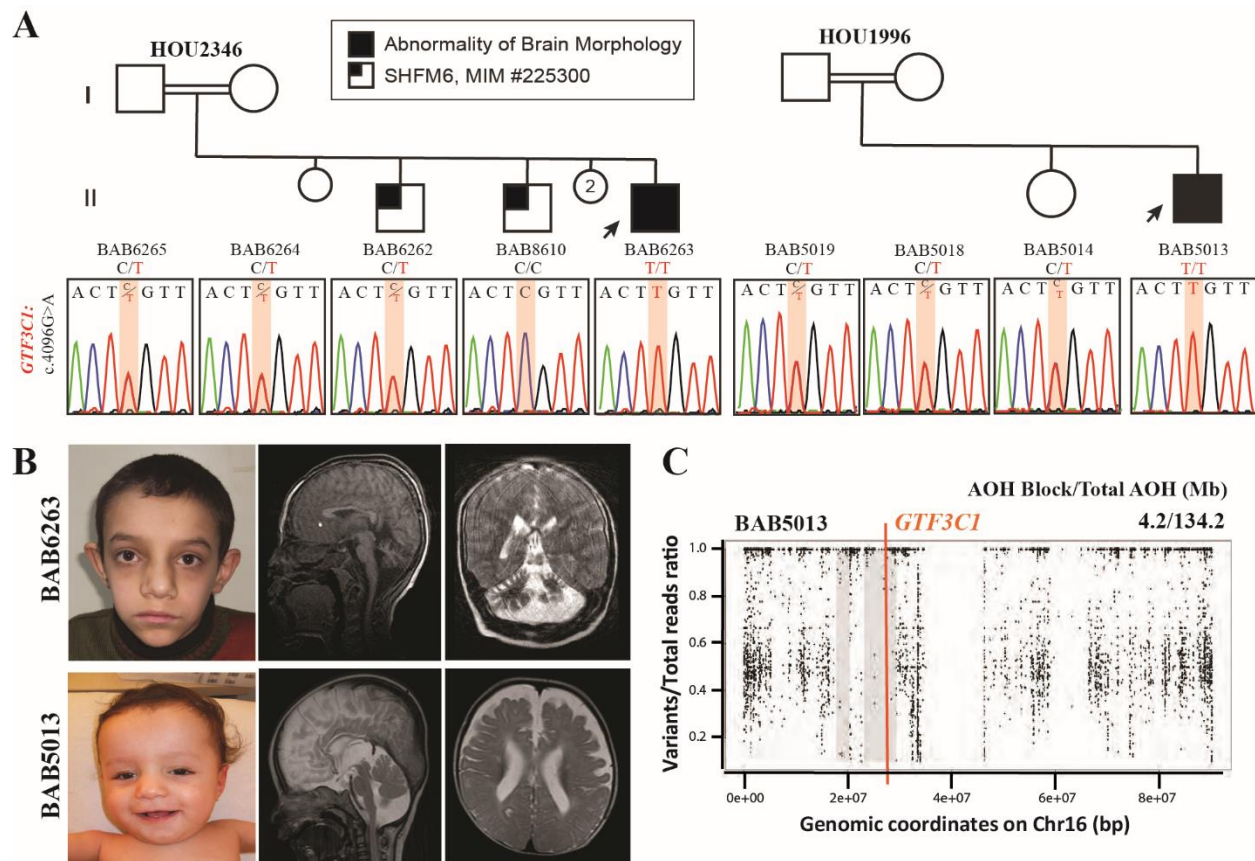

Figure S3. Pedigree, clinical photo, and segregation analysis of a homozygous missense variant of *GTF3C1* in two unrelated Turkish families. (A) Left: pedigree of HOU2346 with reported parental consanguinity. Missense variant (*GTF3C1*: c.4096G>A, p.E1366K) profiled by Sanger dideoxy sequencing, segregated with brain malformation in accordance with Mendelian

expectations for a recessive trait. Segregation was previously published by our group as the right pedigree of HOU1996 and the same variant (*GTF3C1*: c.4096G>A, p.E1366K). (B) The top panel shows the facial image of BAB6263, followed by brain MRIs demonstrating cerebellar atrophy. The bottom panel displays the facial appearance of BAB5013 and evidence of cerebral atrophy seen in the brain MRIs. (C) The AOH plot for BAB5013 demonstrates that the location of the missense variant allele of *GTF3C1* (marked by a red vertical line) is within a 4.2 Mb AOH block (denoted by the gray zone) on chromosome 16 with total AOH as 134.2 Mb.

Figure S4

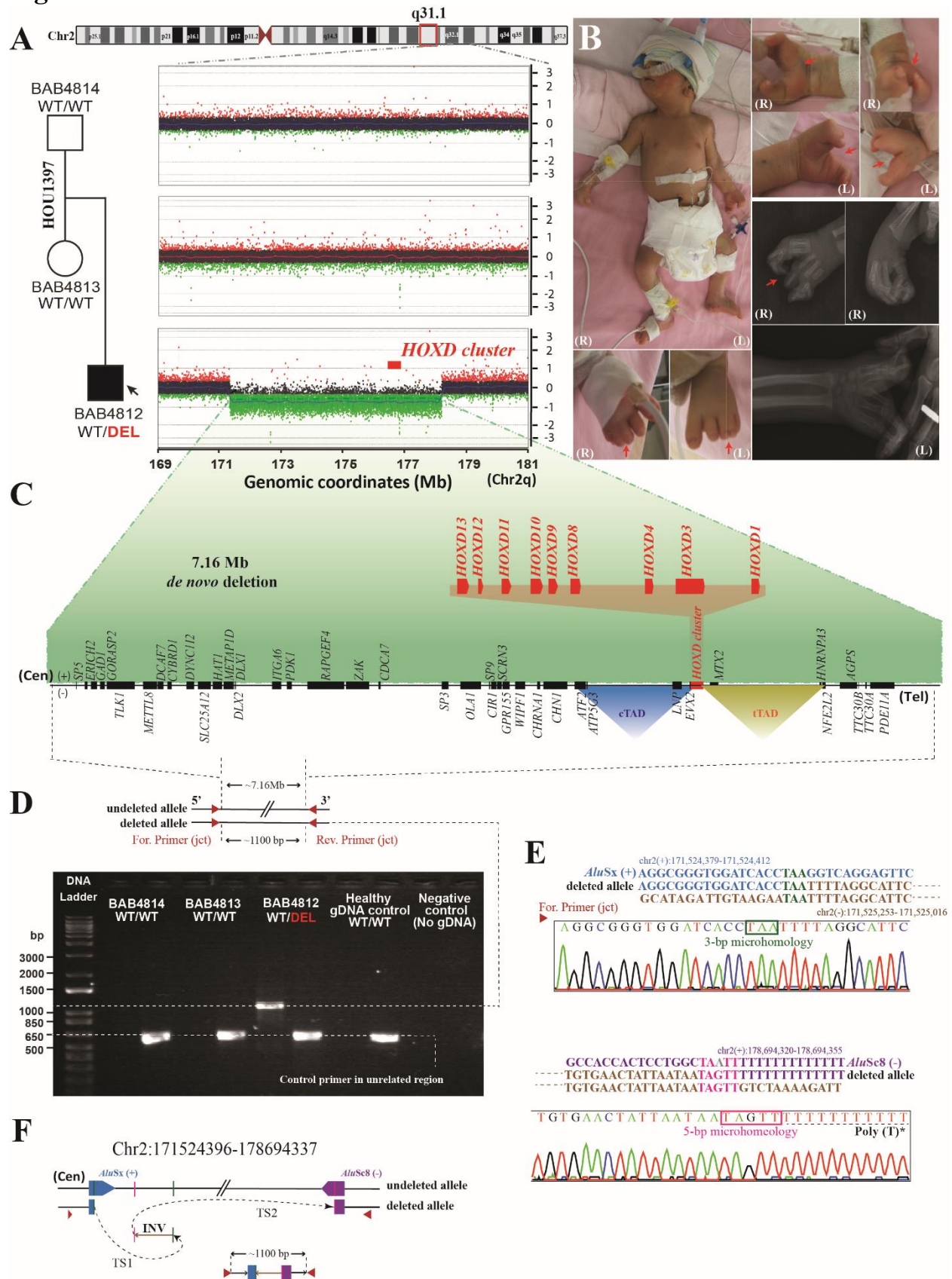

Figure S4. *De novo* heterozygous deletion mediated by AAMR encompassing the entire *HOXD* territory causing a syndromic SHFM5 phenotype.

- A. Pedigree structure of family HOU1397, and a customized HD-aCGH designed to interrogate Chr2q31.1 of the proband and parents, confirmed that this 7.16 Mb heterozygous deletion observed in the proband (area marked by green shade) was a *de novo* mutational event (both parents are ‘wild type’ normal).
- B. Clinical images and radiographs of proband BAB4812 show the bilateral split hands (missing 3rd and 4th digit rays).
- C. The illustration shows a magnified view of the genomic architecture and depicts the gene name/symbol, length, orientation, and position of each disrupted gene within this large deletion (Cen: centromere; Tel: telomere). The red rectangle highlights the linear structure of the *HOXD* gene cluster with two flanking triangles demarcating each side, shaping the two large adjacent gene deserts with a regulatory archipelago in which the regulatory TADs of the *HOXD* cluster were discovered.
- D. The diagram shows the positions of a flanking primer pair designed to capture the deleted allele by producing an amplicon size of ~1.1 kb encompassing the approximate breakpoint location estimated from aCGH data. A positive control primer pair was applied for PCR of an ~650 bp unrelated genomic region. Below the diagram is the 1% agarose electrophoresis of the PCR products. Only the proband DNA shows a specific band of ~1.1 kb, representing the deleted allele.
- E. Sanger sequencing from the ~1.1 kb PCR product of the proband revealed that the deleted allele recombinant junction encompasses three distinct sequences: two flanking *Alu* elements (*AluSx/AluSc8*) in the opposite orientation with an inverted sequence (~300bp downstream to the 5' *AluSx*+) inserted in the middle. The first breakpoint junction encompasses a 3-bp microhomology (three sequence above the Sanger sequencing trace from top to bottom denote the reference *AluSx*+ reference (colored in blue), the proband ‘chimeric’ sequence, and the inverted sequence (colored in brown). The second breakpoint junction (bottom), highlighting a 5-bp microhomeology (three sequence above the sequencing trace from top to bottom), denotes the *AluSc8*- reference sequence (colored in purple), the proband ‘chimeric’ sequence, and reference inverted sequence colored in brown.
- F. A schematic diagram elucidates the hypothesized formation of this DEL-INV-DEL structure

driven by an MMBIR-based, two-step template switching (TS) event mediated by two *Alu* elements in the opposite orientation.

Figure S5

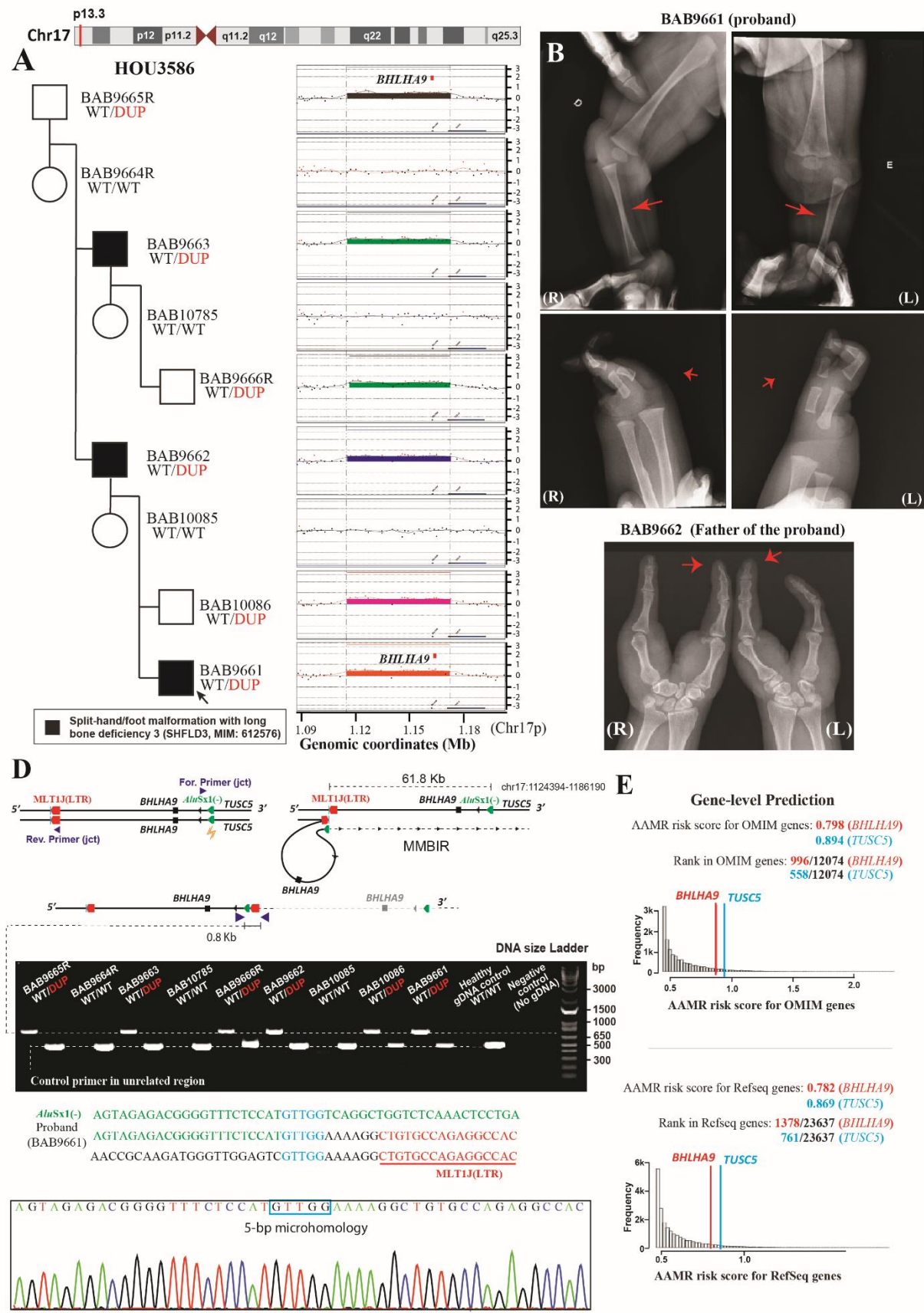

Figure S5: Heterozygous *BHLHA9* duplication in a Brazilian family causing SHFLD3 with reduced penetrance

- A. Pedigree structure of HOU3586 (F35 in da Rocha et al.³⁷) with an HD-aCGH interrogating Chr17p13.3 for each family member. A ~61.8 Kb duplication encompassing the full-length of *BHLHA9* and first exon of *TUSC5* was identified in six individuals following a paternal inheritance mode. However, only three of six duplication allele carriers (BAB9661(F35.1), BAB9662(F35.2) and BAB9663(F35.3)) manifest the SHFLD3 phenotype, consistent with the reduced penetrance frequently ascribed to this locus.
- B. Radiographs of lower and upper limbs of proband BAB9661(F35.1). The top two images demonstrate bilateral tibial hemimelia (marked by red arrows). The lower two images show the bilateral split hand and oligodactyly.
- C. Radiographs of the father BAB9662 (F35.2). There is bilateral split hand malformation with triphalangeal thumbs, oligodactyly and carpal bone abnormalities, including in the right-hand abnormal configuration of the trapezium and trapezoid, narrow capitate and broad and trapezoidal hamate, and the left-hand insertion of the capitate between the scaphoid and trapezoid bones and fusion of the hamate, triquetrum and pisiform bones.
- D. A schematic diagram shows the hypothetical model of *BHLHA9* tandem duplication formed by an MMBIR-based genomic rearrangement. A single-strand break, a one-ended double-stranded DNA molecule (oeDNA) likely generated from a collapsed fork, occurred in an *AluSx1*- element (yellow mark) and introduced a microhomology (marked by blue bar)-mediated template switching into a 61.8 kb upstream region. A primer set was designed to capture the tandem duplicated allele by PCR, producing an amplicon size of ~0.8 kb encompassing the approximate breakpoint location. Control primer pair was applied on a ~500 bp unrelated genomic region. Below the diagram is the 1% agarose electrophoresis of the PCR product; the specific band of ~800bp is the tandem duplicated allele captured only in six duplication carriers. Sanger sequencing (bottom) from the ~800bp PCR product of the proband revealed a 5-bp microhomology. Three sequences above the Sanger sequencing trace from top to bottom denote the 3' *AluSx1*+ reference (colored in green), the proband 'chimeric' sequence, and the 5' reference sequence (adjacent downstream LTR colored in red).
- E. Gene-level prediction from AluAluCNVPredictor shows the machine learning elevated

AAMR relative risk score of *BHLHA9* and *TUSC5* in OMIM genes (0.798 and 0.894) and in RefSeq genes (0.782 and 0.869), respectively. This score represents a human genome genomic instability risk.

Figure S6

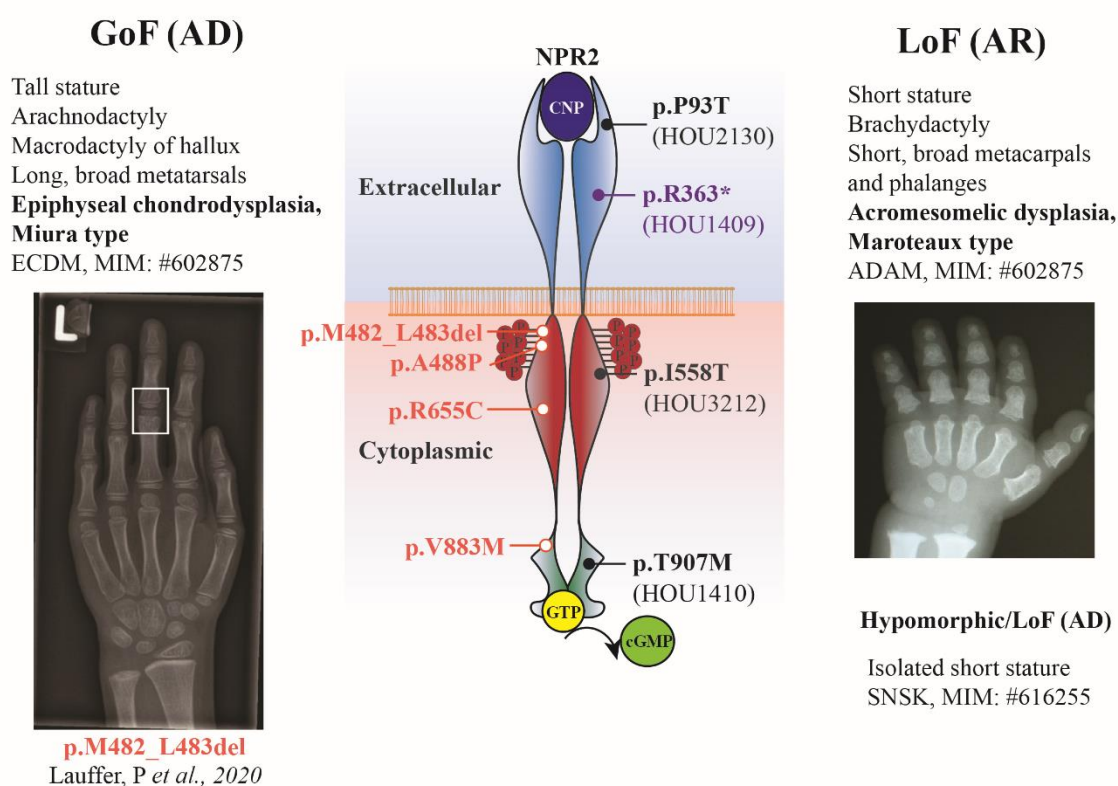

Figure S6. An illustration shows the “mirror trait” produced by the allelic series of *NPR2*. This diagram shows a typical model for a ‘mirror trait’ denoted as the opposite extremes of the phenotype observed in association with LoF versus GoF alleles. Biallelic LoF variants of *NPR2* reported in this study result in AMDM primarily due to dysregulation of CNP-NPR2-cGMP signaling homeostasis. The patient affected by AMDM shows short stature and acromesomelia featured by short and broad metacarpals and phalanges. However, the mirror opposite disease trait was observed in the patient who carried a heterozygous GoF variant and manifested tall stature, arachnodactyly, extra epiphyses, and elongated metacarpals and phalanges due to elevated hormonal catalytic activity.

Figure S7

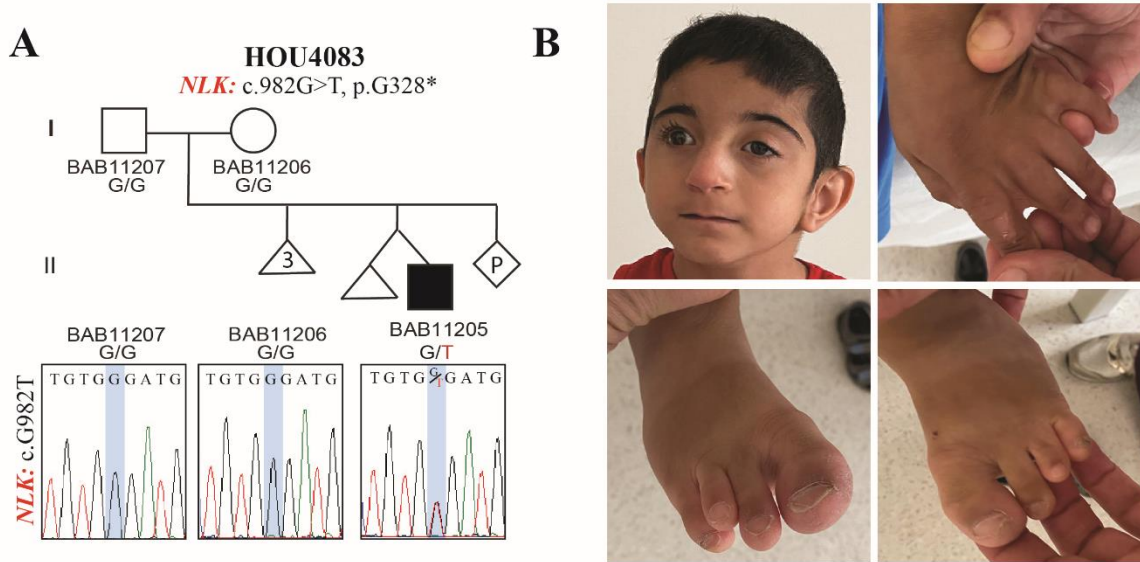

Figure S7. Pedigree, clinical photo, and segregation analysis of a *de novo* nonsense variant of *NLK* in a Turkish family. (A) Pedigree of HOU4083 and variant information was reported from our group. Below the pedigree is the Sanger sequencing confirmation showing the *de novo* nonsense variant (*NLK*: c.982G>T, p.G328*) is only present in the proband (BAB11205). (B) Clinical photos of BAB11205 manifest unilateral left microphthalmia, 2nd/3rd finger syndactyly on the left hand, and bilateral foot oligodactyly.

SUPPLEMENTAL MATERIAL AND METHODS

PCR Primers that were utilized for this study

Gene/Locus	Coordinate (hg19),<br>Nucleotide Change	Forward Primer	Reverse Primer
<i>GLI3</i>	Chr7:42017296_G>T	TTTCCAGTCCCACCTA<br>GGAA	AAAAGCTGCTGACC<br>CTTGAA
<i>GTF3C1</i>	Chr16:27492500_C>T	CTGAAGGACCAATGG<br>GAGAA	CAGGTACCCCAGTT<br>TCCTGA
<i>HOXD13</i>	Chr2:176958241_A>T	TACAGCAGAATGCGC<br>TCAAG	CACAACTCCCCTC<br>CCAAGT
Junction_Chr17p13.3<br>(HOU3358)	Chr17:1164471-<br>1239336; Duplication	GACTGCACCTCATCT<br>CACTAATCA	CATCCTCCATCATG<br>AACAAGAG
Junction_Chr17p13.3<br>(HOU3586)	Chr17:1124394-<br>1186190; Duplication	CTTTTCCCGTGCTCTT<br>TCTG	GTCTCCCAGGTTCA<br>TGCAAT
Junction_Chr2q31.1	Chr2:171524396-<br>178694337; Deletion	CCCCATTGCCTTGTTA<br>ATTG	TCACTCCAGTTGGC<br>TGTGAC
<i>NPR2</i>	Chr9:35802243_T>C	CCCACCATTTCATCCT<br>GTCT	GCAAATGTTGGGAG<br>GGTCTA
<i>NPR2</i>	Chr9:35792682_C>A	GCACTAGCTGTGGAG<br>GCTCT	TGAAGTGCCCGTGT<br>AGTGTC
<i>NPR2</i>	Chr9:35800118_C>T	CATCAAGTCTTGGCT<br>GTGGA	GTGGGCAACTCTGC<br>CATATT
<i>NPR2</i>	Chr9:35808513_C>T	TCACCAGCCTCTGTC<br>CTCTT	CAGACTGGCCCTTT<br>GAGAAG
<i>WNT10B</i>	Chr12:49360306_TG>T	CAGCACATAGCAGCA<br>CCAGT	CAGCACATAGCAGC<br>ACCAGT
