# Supplementary material for: Developmental genomics of limb malformations: Allelic series in association with gene dosage effects contribute to the clinical variability
Source: HGG Adv. 2022 Aug 4;3(4):100132. doi: 10.1016/j.xhgg.2022.100132 (PMC9403727; doi:10.1016/j.xhgg.2022.100132)
Supplement: Document S2. Article plus supplemental information [file mmc2.pdf]

Developmental genomics of limb malformations: Allelic series in association with gene dosage effects contribute to the clinical variability

Ruizhi Duan,¹ Hadia Hijazi,^{1,22} Elif Yilmaz Gulec,² Hatice Koçak Eker,³ Silvia R. Costa,⁴ Yavuz Sahin,⁵ Zeynep Ocak,⁶ Sedat Isikay,⁷ Ozge Ozalp,⁸ Sevcan Bozdogan,⁹ Huseyin Aslan,⁸ Nursel Elcioglu,^{10,11} Débora R. Bertola,^{4,12} Alper Gezdirici,¹³ Haowei Du,¹ Jawid M. Fatih,¹ Christopher M. Grochowski,¹ Gulsen Akay,¹ Baylor-Hopkins Center for Mendelian Genomics, Shalini N. Jhangiani,¹⁴ Ender Karaca,^{1,19} Shen Gu,^{1,20} Zeynep Coban-Akdemir,^{1,21} Jennifer E. Posey,¹ Yavuz Bayram,^{1,22,23} V. Reid Sutton,^{1,15} Claudia M.B. Carvalho,^{1,24} Davut Pehlivan,^{1,15,16,17} Richard A. Gibbs,^{1,14} and James R. Lupski^{1,14,15,18,25,*}

Summary

Genetic heterogeneity, reduced penetrance, and variable expressivity, the latter including asymmetric body axis plane presentations, have all been described in families with congenital limb malformations (CLMs). Interfamilial and intrafamilial heterogeneity highlight the complexity of the underlying genetic pathogenesis of these developmental anomalies. Family-based genomics by exome sequencing (ES) and rare variant analyses combined with whole-genome array-based comparative genomic hybridization were implemented to investigate 18 families with limb birth defects. Eleven of 18 (61%) families revealed explanatory variants, including 7 single-nucleotide variant alleles and 3 copy number variants (CNVs), at previously reported “disease trait associated loci”: *BHLHA9*, *GLI3*, *HOXD* cluster, *HOXD13*, *NPR2*, and *WNT10B*. Breakpoint junction analyses for all three CNV alleles revealed mutational signatures consistent with microhomology-mediated break-induced replication, a mechanism facilitated by *Alu/Alu*-mediated rearrangement. Homozygous duplication of *BHLHA9* was observed in one Turkish kindred and represents a novel contributory genetic mechanism to Gollop-Wolfgang Complex (MIM: 228250), where triplication of the locus has been reported in one family from Japan (i.e., $4n = 2n + 2n$ versus $4n = 3n + 1n$ allelic configurations). Genes acting on limb patterning are sensitive to a gene dosage effect and are often associated with an allelic series. We extend an allele-specific gene dosage model to potentially assist, in an adjuvant way, interpretations of interconnections among an allelic series, clinical severity, and reduced penetrance of the *BHLHA9*-related CLM spectrum.

Introduction

Vertebrate limb development is a sophisticated, patterned process with the limbs formed in the early embryonic stages because of underlying mesodermal-ectodermal cellular interactions. Orchestrated by complex “fine-tuned” gene dosage expression, tissue/cell gradients of

developmental factors or signal molecules are formed through inductive interactions between mesoderm/ectoderm tissues and oscillation of several signal transduction pathways.^{1–3} 3D axes (anterior/posterior [A/P], dorsal/ventral [D/V], and proximal/distal [P/D]) are interconnected to coordinate homeostatic cell population/tissue/organismal development of the apical ectodermal ridge

¹Department of Molecular and Human Genetics, Baylor College of Medicine, Houston, TX, USA; ²Department of Medical Genetics, School of Medicine, Istanbul Medeniyet University, Istanbul, Turkey; ³Department of Medical Genetics, Konya City Hospital, Konya, Turkey; ⁴Human Genome and Stem Cell Research Center, Institute of Bioscience, Universidade de São Paulo, São Paulo, Brazil; ⁵Medical Genetics, Genoks Genetics Center, Ankara, Turkey; ⁶Department of Medical Genetics, Faculty of Medicine, Istinye University, Istanbul, Turkey; ⁷Department of Pediatric Neurology, Faculty of Medicine, Gaziantep University, Gaziantep, Turkey; ⁸Department of Medical Genetics, Adana City Training and Research Hospital, Adana, Turkey; ⁹Department of Medical Genetics, Faculty of Medicine, Cukurova University, Adana, Turkey; ¹⁰Department of Pediatric Genetics, School of Medicine, Marmara University, Istanbul, Turkey; ¹¹Eastern Mediterranean University Medical School, Magosa, 10 Mersin, Turkey; ¹²Genetics Unit, Instituto da Criança do Hospital das Clínicas da Faculdade de Medicina, Universidade de São Paulo, São Paulo, Brazil; ¹³Department of Medical Genetics, Basaksehir Cam and Sakura City Hospital, Istanbul, Turkey; ¹⁴Human Genome Sequencing Center, Baylor College of Medicine, Houston, TX, USA; ¹⁵Texas Children's Hospital, Houston, TX, USA; ¹⁶Section of Pediatric Neurology and Developmental Neuroscience, Department of Pediatrics, Baylor College of Medicine, Houston, TX, USA; ¹⁷Jan and Dan Duncan Neurological Research Institute at Texas Children's Hospital, Houston, TX, USA; ¹⁸Department of Pediatrics, Baylor College of Medicine, Houston, TX, USA

¹⁹Present address: Sanford Molecular Genetics Laboratory, Sioux Falls, SD, USA

²⁰Present address: School of Biomedical Sciences, Faculty of Medicine, Chinese University of Hong Kong, HKSAR, China

²¹Present address: Human Genetic Center, Department of Epidemiology, Human Genetics, and Environment Sciences, School of Public Health, The University of Texas Health Science Center at Houston, Houston, TX, USA

²²Present address: Division of Genomic Diagnostics, Department of Pathology and Laboratory Medicine, Children's Hospital of Philadelphia, Philadelphia, PA, USA

²³Present address: Perelman School of Medicine, University of Pennsylvania, Philadelphia, PA, USA

²⁴Present address: Pacific Northwest Research Institute, Seattle, WA, USA

²⁵Lead contact

*Correspondence: jlupski@bcm.edu

<https://doi.org/10.1016/j.xhgg.2022.100132>

© 2022 The Author(s). This is an open access article under the CC BY license (<http://creativecommons.org/licenses/by/4.0/>).

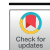

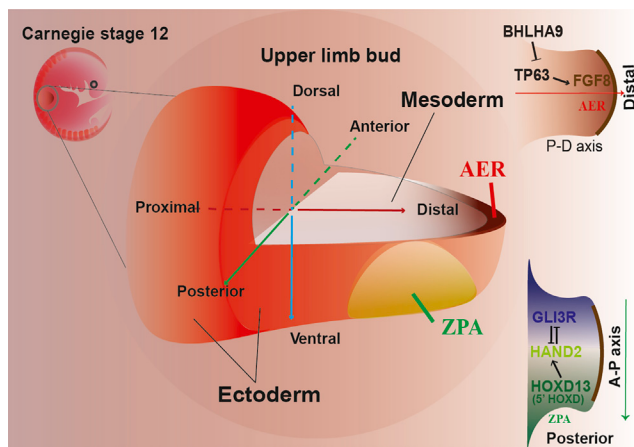

Figure 1. A schematic of embryonic upper limb development

An illustrative drawing describes a human embryo at Carnegie stage 12 (top left; approximately 4 weeks after conception), when the limb bud forms as a ventrolateral bulge and begins to emerge outwardly. Also shown is a magnified illustration depicting the 3D patterning of the upper limb bud and a dissected view of the anatomical position between ectoderm and mesoderm. The P/D, A/P, and D/V axes are labeled with different colors. The AER is labeled at the brown epithelial region, ZPA is shown as a gradient-based yellow zone. Bottom right: illustration of a mutual genetic antagonism between the short isoform of GLI3 (GLI3R) and HAND2 that is required to establish a “pre-patterning” of the A/P axis prior to formation of the SHH-ZPA gradient. Top right: P/D elongation is regulated by many signaling elements, including FGF8, TP63, and BHLHA9.

(AER), zone of polarizing activity (ZPA), and limb bud development (Figure 1). Disruption of gene action or gene regulation for genes executing tissue proliferation, differentiation, cell migration, or stratification for each axial patterning plane can cause congenital limb malformation (CLM).

CLM represents a broad spectrum of prenatal developmental defects, including limb reduction, appendicular skeletal anomalies, and/or abnormalities in the number, length, anatomical positioning, and anatomy of the digits.⁴ CLMs affect approximately 1 in 500 newborns, with occurrence as a deformation, disruption, or isolated developmental event (i.e., malformation), or it can be a manifestation of an endophenotype of a rare Mendelian disease trait.^{5,6} In addition to extensive interfamilial and intrafamilial clinical and genetic heterogeneity, incomplete penetrance and variable expressivity have been frequently highlighted in CLM studies; these genetic features are among some of the inherent challenges of concluding molecular diagnosis(es) for children and families manifesting CLM and for genetic diagnoses from unphased genomic information.

During the past decade, elucidation of the molecular etiology of CLM has been facilitated by advances in high-throughput family-based genomics, rare variant allele studies, computational tools, and population variation studies, driving the molecular diagnostic “solved rate” of a random CLM-related cohort to be reported with a range of 10%–35% in different populations.^{7–11} To date, more than 150 “disease trait genes or loci” have been attributed

to CLM in affected individuals and families. A diverse mutational landscape comprised of single-nucleotide variant (SNV) alleles, small insertion or deletion (indel) events, simple sequence repeat expansions, and locus copy number variant (CNV) alleles involving coding or non-coding regulatory regions has been elucidated.^{8,12,13}

Despite the continuing efforts of genome-wide screening and novel molecular etiological insights provided for CLM, many candidate genes/loci remain uncharacterized or under-characterized, or allelic series at a locus are not explored. Some clinically well-described CLMs with numerous case reports published through decades of research still lack a clear underlying contributing genetic variant explanation: examples include Gollop-Wolfgang complex (GWC; MIM: 228250) and fibular aplasia, tibial campomelia, and oligosyndactyly (FATCO) syndrome (MIM: 246570). Recurrent CLM-associated CNVs and other variant types are often poorly elucidated and mechanistically enigmatic from a mutational mechanism standpoint, whether as *de novo* allelic variants associated with a sporadic congenital anomaly or contributing as a recently arising mutation in clan ancestors.¹⁴ Contributions to a biallelic variant state are not always genetically clear as to when homozygosity results from identity by state (IBS; identical alleles/genomic segments do not share a common ancestor) versus identity by descent (IBD; identical alleles/genomic segments inherited from a common ancestor without intervening recombination). In this study, we implemented family-based genomics and rare variant analyses as a molecular “entry point” to decipher the biology, mutational features, and mutational mechanisms underlying the genetic and developmental basis of CLM.

Methods and materials

Sample preparation

Prior to enrollment for genomic studies, the phenotype of each affected individual was initially investigated by local clinical geneticists and re-evaluated by physicians, including pediatricians, pediatric neurologists, and medical geneticists, in our research genomics group. To reduce the clinical heterogeneity, the recruited cases were primarily those with phenotypic findings involving the appendicular skeleton, focusing on limb anomalies as their major structural morphogenesis defects. Peripheral blood from each registered individual and associated family members was collected locally in EDTA tubes and shipped to our lab for genomic DNA extraction using standard protocols.

Exome sequencing analysis and rare variant SNV prioritization

Family-based exome sequencing (ES) of all subjects was implemented at the Baylor College of Medicine Human Genome Sequencing Center (BCM-HGSC) under previously described protocols through the Baylor-Hopkins Center for Mendelian Genomics (BHCMG) initiative.^{15–17} In brief, pre-capture library preparation was performed using KAPA HyperPrep Kits, followed by the HGSC-designed core capture protocol (52 Mb, Roche NimbleGen).¹⁸

Paired-end sequencing with multiplex pools was executed using an Illumina HiSeq2000 or NovaSeq6000, generating an average depth of coverage of $\sim 100\times$. Subsequently, post-sequencing data were mapped to the haploid human genome reference sequence assembly (hg19) together with variant calling, post-processing, annotation, and quality control under a standardized HGSC-Mercury pipeline as implemented previously and deployed in the cloud.¹⁹

An unbiased, stepwise computational analysis workflow was employed for SNV filtering, parsing, and prioritization.²⁰ Rare variants were extracted comprehensively based on their minor allele frequency (MAF), calculated collectively from an internal BCM-HGSC exome variant database consisting of personal genomes from $\sim 13,000$ individuals and a variety of public genomic databases, including the Genome Aggregation Database (gnomAD), 1000 Genomes Project (TGP), and the Atherosclerosis Risk in Communities (ARIC) study database. Filtered variants were annotated and prioritized for further study based on MAFs ($<0.5\%$ as threshold) and pathogenicity prediction algorithms, including combined annotation-dependent depletion (CADD; >20 as deleteriousness)²¹ and the rare exome variant ensemble learner (REVEL; >0.5 as likely damaging)²² for missense variants. Then each annotated variant was further parsed and prioritized via a comprehensive data-mining process based on additional gene- and variant-level information from publicly available databases, including Online Mendelian Inheritance in Man (OMIM; <http://www.omim.org>), PubMed, the Human Gene Mutation Database (HGMD; <http://www.hgmd.cf.ac.uk>), and ClinVar (<https://www.ncbi.nlm.nih.gov/clinvar/>).

Variant validation and segregation analysis

All candidate SNVs and indels were orthogonally validated by Sanger dideoxy sequencing of the target region amplified using conventional PCR (HotStar Taq, QIAGEN). Familial segregation analysis was examined in accordance with Mendelian expectations on all identified candidate variants alleles, with potential penetrance evaluated by physical examination and radiographs.²³

CNV identification and analysis

Large CNVs (>3 -exon deletion/duplication) were initially called *in silico* from exome data via several read-depth-based algorithms, including exome-hidden Markov model (XHMM) and HMZDelFinder for smaller homozygous deletion.^{24,25} Using a family-based genomics approach and rare CNV filtering, potential pathogenic CNVs were detected (families HOU1397, HOU3358, and HOU3586).

The genomic architecture of each potential pathogenic CNV was experimentally investigated in affected individuals and selected family members by customized high-definition array-based comparative genomic hybridization (HD-aCGH), using arrays designed by implementing the eArray system (<https://earray.chem.agilent.com/suredesign/>) and array platforms manufactured by Agilent (AMAD: 086724). Microarray protocols, including DNA digestion, probe labeling, gender-matched hybridization, and post-washing with minor modifications, were performed as described previously.²⁶ Agilent SureScan and Feature Extraction software was utilized to achieve the image-to-digital transition, with further data analysis and visualization on the Agilent Genomic Workbench.

Genes mapping within genomic intervals potentially susceptible to genomic instability or *Alu/Alu*-mediated rearrangement (AAMR) were investigated by AluAluCNVpredictor for a relative

risk genomic instability index or score, implementing a recently described informatics tool.^{27,28} Approximate genomic intervals of CNVs were determined at single W-C base-pair resolution via breakpoint junction analysis using Sanger dideoxynucleotide sequencing.

Absence of heterozygosity (AOH) and co-efficient of consanguinity analysis

Genomic data evidence for potential consanguinity was investigated quantitatively based on AOH, which can serve as a surrogate measure of runs of homozygosity (ROHs), to profile potential IBD genomic intervals and a coefficient of consanguinity (i.e., the fraction of the assayable human genome haploid reference), as described previously.^{29,30} For an individual genome, ROH genomic regional analyses often provide additional evidence for variant prioritization, serving as an adjuvant tool to probe the genetic and allelic genomic architecture and autozygous intervals (IBD). In this study, AOH data visualization and analysis for individual personal genomes were achieved through BafCalculator, an in-house-developed bioinformatics method for B allele frequency computation from unphased ES-variant data.³¹

Study approval

Eighteen families with clinical diagnoses of CLM were recruited into the BHCMG under an institutional review board (IRB; H-29697)-approved study.¹⁵ All participating subjects signed informed consent regarding data sharing and publication of medical information and photographs.

Results

Characteristics of study subjects and family-based genomic analyses

From 2012–2020, 18 families with rare congenital defects involving limbs or the appendicular skeleton were enrolled into the BHCMG, now the BCM-Genomics Research to Elucidate the Genetics of Rare (BCM-GREGoR) disease consortium, with a mandate to “solve the unsolved.” Using family-based genomics and rare variant analyses, 7 SNV and 3 CNV alleles were identified as plausible molecular diagnostic etiologies for 11 of the 18 studied families, interpreted by American College of Medical Genetics and Genomics (ACMG)-based variant classification criteria (Table 1 and Table 2).^{32,33} Most gene products involve the P/D and A/P axial formation of the limbs.

Intrafamilial phenotypic variability was commonly observed in the studied families and associated with reduced penetrance and variable expressivity (Table 2). Sometimes, variability in presentation manifests as unilateral or asymmetric anatomical patterns (e.g., left/right, upper/lower limbs) for an individual, with malformation observed specifically in an asymmetric pattern on only the left/or right side or upper/lower limbs.

Novel variant identification involving genes contributing to A/P axis patterning

Polydactyly preaxial type IV (PPD4; MIM: 174700), alternatively known as polysyndactyly, represents manifestations

Table 1. Summary of identified SNVs and CNVs in the cohort

	Family	Country of origin	Consanguinity	Gene/locus	Variant type	Variant (GRCh37\hg19)	Zygosity	Allele count (gnomAD)	CADD score (Phred-scale)	Conservation, (phyloP20way_mammalian)	ACMG variant classification criteria	
											Evidence category/section	Classification
SNV	HOU2084	Turkey	no	<i>GLI3</i>	nonsense	NM_000168:<br>c.1673C>A (p.Ser558*)	Htz	0 Htz–0 Hmz	N/A	1.048	PVS1, PM2, PP1, PP3	pathogenic
	HOU3022	Turkey	no	<i>HOXD13</i>	missense	NM_000523:<br>c.623A>T (p.Asp208Val)	Htz	0 Htz–0 Hmz	28.8	0.977	PM2, PP2, PP3	uncertain significance
	HOU3212	Turkey	yes (1 ^O cousins)	<i>NPR2</i>	missense	NM_003995:<br>c.1673T>C (p.Ile558Thr)	Hmz	2 Htz–0 Hmz	25.5	0.997	PS1, PM2, PP1, PP3	likely pathogenic
	HOU2130	Turkey	yes (1 ^O cousins)	<i>NPR2</i>	missense	NM_003995:<br>c.277C>A (p.Pro93Thr)	Hmz	0 Htz–0 Hmz	24.4	1.026	PM2, PP1, PP2, PP3, PP4	likely pathogenic
	HOU1409	Turkey	yes (1 ^O cousins)	<i>NPR2</i>	nonsense	NM_003995:<br>c.1087C>T (p.Arg363*)	Hmz	1 Htz–0 Hmz	N/A	N/A	PVS1, PM2, PP1, PP3, PP4	pathogenic
	HOU1410	Turkey	yes (1 ^O cousins)	<i>NPR2</i>	missense	NM_003995:<br>c.2720C>T (p.Thr907Met)	Hmz	0 Htz–0 Hmz	32	1.048	PS1, PM2, PP1, PP3	likely pathogenic
	HOU2346<br>HOU3360	Turkey	yes (1 ^O cousins)	<i>WNT10B</i>	frameshift	NM_003394:<br>c.741delC (p.Cys247*)	Hmz	0 Htz–0 Hmz	N/A	N/A	PVS1, PM2, PP1, PP3, PP4	pathogenic
CNV	HOU1397	Turkey	no	2q31.1	deletion	chr2:171,524,396–178,694,337;<br>7.16 mb	Htz				1A, 2A, 3C, 5A	pathogenic
	HOU3586 (F35 ^a)	Brazil	no	17p13.3	duplication	chr17:1,124,394–1,186,190,<br>61.8 kb	Htz				1A, 2A, 3A, 4H, 4L, 5D	pathogenic
	HOU3358	Syria	yes (1 ^O cousins)	17p13.3	duplication	chr17:1,164,471–1,239,336,<br>74.9 kb	Hmz				1A, 2A, 3A, 4L, 5D	pathogenic

Hmz, homozygous; Htz, heterozygous; N/A, not available; CADD, combined annotation-dependent depletion.

^aThis family was published previously as family 35 (F35) by da Rocha et al.⁵³

Table 2. Summary of clinical information in 18 unrelated families with phenotypic findings on limbs and appendicular skeletons

	Family	Individual	Dz, OMIM	Phenotype of upper limbs		Phenotype of lower limbs		Other phenotypes and information
				Left	Right	Left	Right	
A/P	HOU2084	BAB5343	polydactyly preaxial type 4; MIM: 174700		syndactyly between third and fourth digits, postaxial polydactyly	bilateral preaxial polydactyly of halluces, syndactyly involving second and third toes		Solved
	HOU3022	BAB8289	synpolydactyly type 1; MIM: 186000		mesoaxial polydactyly and syndactyly on the fourth digit	postaxial polydactyly on fifth toe, syndactyly involving fourth toe		
P/D	HOU3212	BAB8908	acromesomelic dysplasia, Maroteaux type (AMDM); MIM: 602875			bilateral macrodactyly of halluces with brachydactyly of toes		
	HOU2130	BAB5498	AMDM; MIM: 602875	brachydactyly; short, broad metacarpals/metatarsals and phalanges; restricted elbow extension				<i>pectus excavatum</i> , micromelia with predominant rhizomelia
		BAB5499						short, bowed forearm; platyspondyly; thoracic kyphosis; and congenital hip dislocation
	HOU1410	BAB3611	AMDM; MIM: 602875	Brachydactyly; short, broad metacarpals/metatarsals and phalanges; hyperextensibility of metacarpals; restricted elbow extension				short and thick humeri, platyspondyly
		BAB3612		Brachydactyly; short, broad metacarpals/metatarsals and phalanges; tubular bone shortness				short and thick humeri, platyspondyly, flattened acetabulum and <i>coxa vara</i>
	HOU1409	BAB3606	AMDM; MIM: 602875	Brachydactyly; short, broad metacarpals/metatarsals and phalanges				short and thick humeri, platyspondyly
		BAB3607						
	HOU2346	BAB6262	split-hand/foot malformation (SHFM) type 6; MIM: 225300		syndactyly involving third and fourth digit	bilateral split foot and syndactyly		
		BAB8610		joint contracture on second digit				
	HOU3360	BAB9281		bilateral split hand, oligodactyly, and syndactyly		bilateral split foot and syndactyly		
		BAB9286						
		BAB9316						
	HOU1397	BAB4812	SHFM5; MIM: 606708	bilateral split hand, oligodactyly, and syndactyly		bilateral syndactyly involving third and fourth toes		see Supplemental Note
	HOU3586 (F35 ¹)	BAB9661 (F35-1)	SHFM with long bone deficiency type 3; MIM: 612576	bilateral split hand		bilateral tibial aplasia		
		BAB9662 (F35-2)		bilateral split hand, triphalangeal thumbs		bilateral tibial aplasia		
	HOU3358	BAB9273	GWC; MIM: 228250			bilateral femur bifurcation, absent of tibia and monodactyly		

(Continued on next page)

Table 2. Continued

Family	Individual	Dz, OMIM	Phenotype of upper limbs		Phenotype of lower limbs		Other phenotypes and information	
			Left	Right	Left	Right		
N/A	HOU1558	BAB4015	N/A		preaxial polydactyly (seven digits) with duplicated hallux and second toe		sacral dimple, age-appropriate neuromotor development, secundum type atrial septal defect, normal brain MRI, abdominal ultrasound and eye examination; no consanguinity between parents, and our child is the first child of the couple	unsolved
	HOU1780	BAB4381	N/A			anteriorly placed fifth toe	5-day-old male infant born at 33rd gestational week with a birth weight of 1,800 g; atresia of anus and duodenum, hypospadias, high and narrow palate, atypical hair color; karyotype and cranial ultrasound unremarkable; died because of multiple medical comorbidities; parents were first-degree cousins	
	HOU1841	BAB4486	fibular aplasia, tibial campomelia, and oligosyndactyly (FATCO) syndrome; MIM: 246570			unilateral (right) fibular aplasia, tibial bowing and hypoplasia (campomelia), rudimentary right foot with oligosyndactyly	parents are unrelated, he has one healthy sister	
	HOU2133	BAB5510		oligosyndactyly with micromelia on second and third digits	oligodactyly with absence of fifth ray	bilateral fibular aplasia, tibial campomelia, and oligosyndactyly	case report was previously published by Sezer et al. ⁵⁹	
	HOU2245	BAB5902	N/A	bilateral camptodactyly with left hand oligodactyly, single palmar crease		brachydactyly on fourth and fifth toes	short stature, facial dysmorphism, narrow thorax, patent ductus arteriosus, ID, and splenomegaly	
	HOU4419	BAB11857	N/A	bilateral upper limb deformity with absence of thumbs			normal weight, height, and head circumference; epilepsy; no consanguinity between parents	
	HOU4083	BAB11205	N/A	syndactyly between second and third digits		bilateral oligodactyly, short and bowing femora	see Supplemental Note	candidate (NLK: c.982C>T [p.Gly328*])

Dz, disease; A/P, anterior/posterior; P/D, proximal-distal.

^aThis family was published previously as family 35 (F35) by da Rocha et al.⁵³

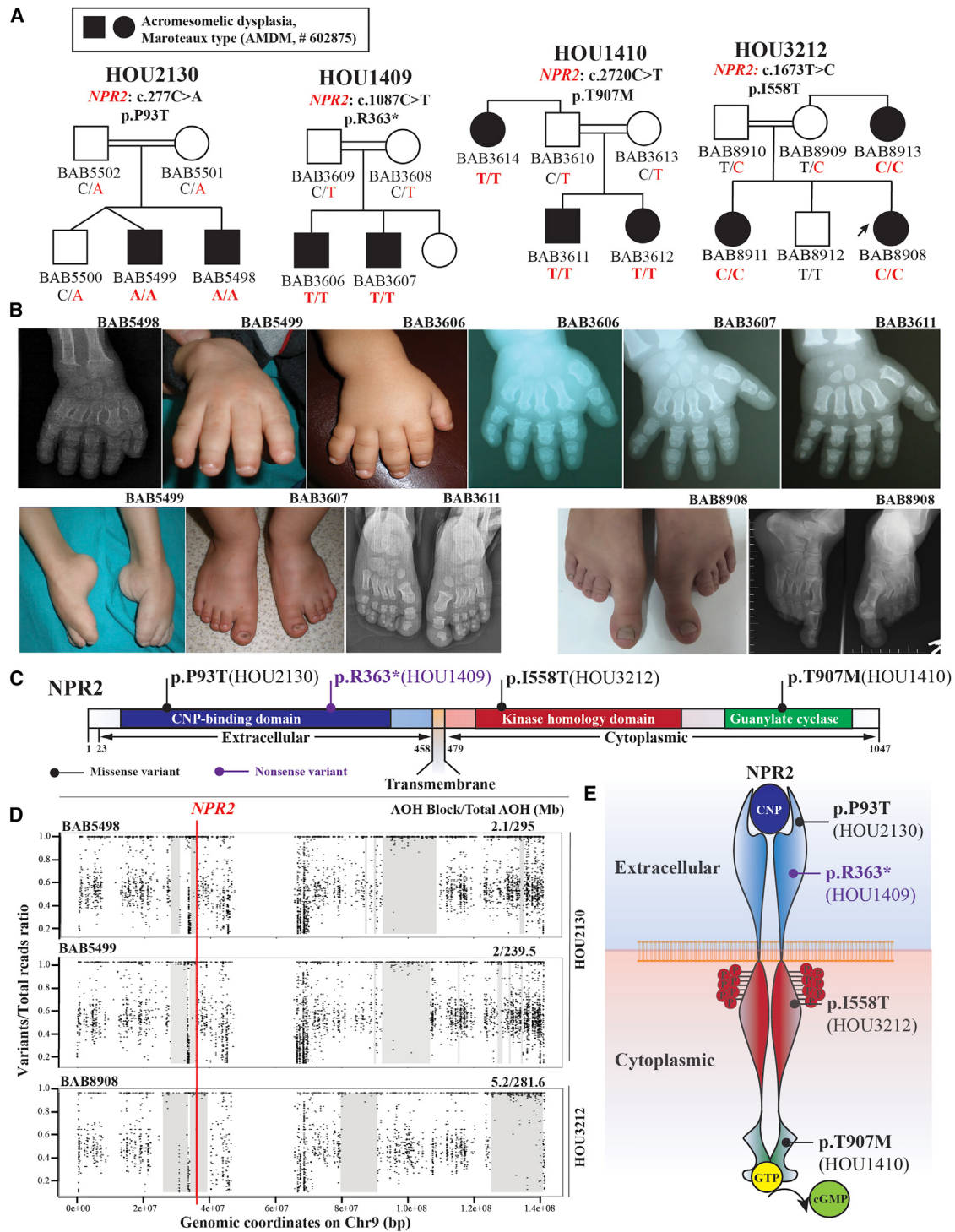

Figure 2. Biallelic disruption of *NPR2* dysregulating longitudinal skeletal growth

(A) Four pedigrees show the segregation of disease-causing variant alleles in each family with AMDM. From left to right: HOU2130 (c.277C>A [p.Pro93Thr]), HOU1409 (c.1087C>T [p.Arg363*]), HOU1410 (c.2720C>T [p.Thr907Met]), and HOU3212 (c.1673T>C [p.Ile558Thr]).

(B) Clinical images and radiographs from selected individuals with *NPR2*-related skeletal dysplasia. The top panel shows brachydactyly, nail hypoplasia, and symmetrically short and broad metacarpals and phalanges. From left to right: BAB5498, BAB5499, BAB3606, BAB3607, and BAB3611. The left bottom panel shows similar findings in the feet. From left to right: BAB5499, BAB3607, and BAB3611. The right bottom panel shows a distinct feature in BAB8908, with macrodactyly of halluces with long and wide metatarsals and phalanges of the great toes as well as brachydactyly of other toes because of short metatarsals and phalanges.

(C) A linear protein structure of the transmembrane receptor *NPR2*, with four identified variant alleles marked in correlated positions (missense variants are labeled as a black line, and a purple line denotes a nonsense variant). Different functional domains and extracellular/cytoplasmic territories of *NPR2* are highlighted in different colors.

(legend continued on next page)

characterized by a bilateral polydactyly presenting preaxially in the thumb/hallux, with variable syndactyly seen in other digits (fingers/toes).^{34,35} In our cohort, PPD4 was observed in multiple members of a Turkish family (HOU2084) as an apparent autosomal dominant (AD) trait (Figures S1A–S1B). ES analyses of three affected individuals revealed a novel heterozygous nonsense variant in the Glioma-associated oncogene (GLI)-Kruppel family member 3 gene, *GLI3* (NM_000168:c.1673C>A [p.Ser558*]). Segregation studies fulfilled Mendelian expectations for a dominant allele with complete penetrance (Figure S1A). This variant allele is predicted by conceptual translation to result in a premature termination codon (PTC) within the N-terminal zinc-finger domain (ZFD) of GLI3; this PTC is predicted to trigger nonsense-mediated decay (NMD), generating a loss-of-function (LoF) variant allele. This interpretation is further supported here by the evidence of a genotype-phenotype observation in the *GLI3*-nonsense variant carriers in family HOU2084, showing only the essential clinical features of PDD4 but no additional findings (Figure S1B).

During limb A/P axial development, a mutual genetic antagonism between GLI3 and heart- and neural crest derivative-expressed protein 2 (HAND2) is required to establish a “pre-patterning” of the A/P axis prior to ZPA formation of the Sonic hedgehog (SHH) gradient (Figure S1C). In contrast to GLI3, HAND2 is expressed in a cell-restricted manner in the posterior mesenchymal region and activated by a direct upstream regulator, Homeobox D13 (HOXD13) (Figure S1C).

Synpolydactyly (SPD1; MIM: 186000) is another “phenotypic combination” with a typical clinical presentation characterized by mesoaxial polydactyly and syndactyly, particularly involving fingers 3/4 and toes 4/5.^{36,37} Trio-ES rare variant analyses of a sporadic proband, BAB8289, who manifested a typical SPD1 phenotype, found a maternally inherited and novel heterozygous missense mutation of *HOXD13* (NM_000523:c.623A>T [p.Asp208Val]) (Figures S1D and S1E). This variant allele is absent in gnomAD and BCM-GREGoR internal databases comprising exome data from ~13,000 individuals. The mother of the proband seems non-penetrant because variant reads are not supportive of mosaicism (variant read to total read ratio as 82/158). This finding in BAB8289 showed that a dominant cause of the disease could be identified in a family from an admixed population with a high coefficient of consanguinity.

Multiple mutation types for pathogenic variant alleles of *HOXD13* segregate with SPD1.³⁸ Aggregated evidence implicates LoF variants of the HOXD13 functional domain driving the typical features of SPD1 and anticipated hap-

loinsufficiency as the most plausible mechanism for the molecular pathogenesis of SPD1.^{39,40} The p.Asp208Val substitution altered an evolutionarily conserved residue outside of, but adjacent to, the homeodomain of HOXD13 (Figure S1D); this variant allele mapped 12 amino acids from a previously reported pathogenic missense variant, p.Gly220Val. As measured by dosage changes, the latter variant allele has been shown experimentally to jeopardize protein stability through aberrant cytosolic accumulation.⁴¹

Biallelic disruption of *NPR2* dysregulating longitudinal skeletal growth

Natriuretic peptide receptor B/guanylyl cyclase B, encoded by *NPR2*, is one of the homodimeric transmembrane hormonal receptors regulating longitudinal skeletal growth during the process of endochondral ossification. Biallelic LoF variants in *NPR2* are known to cause autosomal recessive (AR) acromesomelic dysplasia, Maroteaux type (AMDM; MIM: 602875), an endochondral bone growth disorder characterized by significant short stature, predominantly affecting the forearms and hands.^{42,43} Here we identified two novel and two known pathogenic variants of *NPR2* from four unrelated consanguineous Turkish families (HOU2130, HOU1409, HOU3212, and HOU1410) (Figure 2A). Short stature and acromesomelia, characterized by short and bowed forearms and symmetrically short and broad metacarpals and phalanges with fingernail hypoplasia, are observed in most of the affected individuals (Figures 2B and S2; Table 1B).

In family HOU2130, a novel homozygous missense variant (*NPR2*: c.277C>A [p.Pro93Thr]) was identified through ES analysis of two affected siblings (BAB5498 and BAB5499) with AMDM. This missense variant results in alteration of a conserved residue (p.Pro93Thr) in the C-type natriuretic peptide-binding domain (CNP) of *NPR2* and potentially disrupts the CNP binding affinity. AOH studies mapped the variant to overlapping 2-Mb genomic intervals of AOH/ROH in BAB5498 and BAB5499 encompassing *NPR2*, whereas the total AOH was 295 Mb and 239.5 Mb, respectively, consistent with a clan genomics-derived allele versus a Turkish founder allele (Figures 2A–2D).²⁹

Affected individuals of family HOU1409 and family HOU1410 manifest similar AMDM phenotypes and were directly tested by *NPR2*-targeted Sanger dideoxy sequencing. A novel homozygous nonsense variant of *NPR2* (c.1087C>T [p.Arg363*]) was found in two affected male siblings (BAB3607 and BAB3608) in family HOU1409 segregating with the AMDM phenotype. This

(D) AOH studies on chromosome 9, visualized by B allele frequency data from personal genomes of two affected individuals in family HOU2130 and proband from family HOU3212. The top two panels for family HOU2130 describe a 2.1 Mb (for BAB5498) and 2 Mb (for BAB5499) interval of AOH genomic interval haplotype block (gray shade) surrounding the causative variant of *NPR2* (red vertical line), marked with thick gray rectangles. The bottom panel for HOU3212 denotes a 5.2 Mb AOH interval encompassing the phenotype-associated *NPR2* variant allele identified in individual BAB8908.

(E) An illustrative drawing showing the *NPR2* homodimer and process of CNP-induced cGMP production. Shown are four identified variant alleles located in different functional domains, and each side of transmembrane territories.

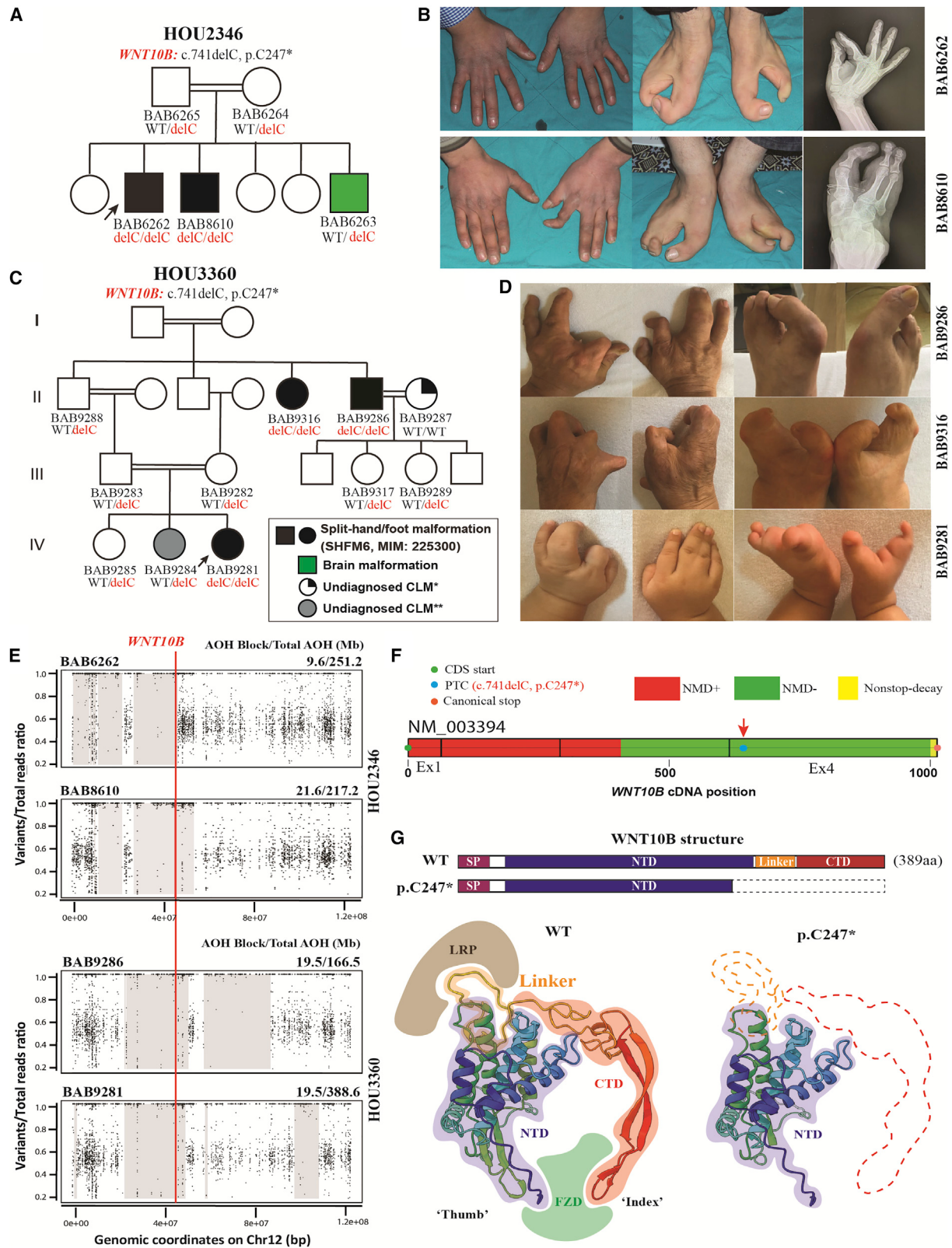

Figure 3. A novel homozygous NMD-escaping frameshift variant of *WNT10B* driving the onset of SHFM6 in two unrelated families from Turkey

(A) Pedigree structure with allelic information, showing segregation of the novel indel variant that leads to a nonsense allele (*WNT10B*: c.741delC [p.Cys247*]) in family HOU2346. Proband BAB6262 (black arrow) was born from a first-cousin mating. He has two male siblings. One brother, BAB8610, is affected by same SHFM phenotype; the other brother, BAB6263 (shaded green on the pedigree), was diagnosed with cerebral atrophy but no limb malformation.

(legend continued on next page)

novel stop-gain variant allele resulted in a PTC in exon 4 (total 21 exons) of *NPR2* and was predicted to be subject to NMD (Figure 2C). In family HOU1410, we identified a known pathogenic homozygous missense variant allele (*NPR2*, c.2720C>T [p.Thr907Met]) in two affected siblings (BAB3611 and BAB3612) and their affected paternal aunt (BAB3614). This missense variant altered a conserved residue mapping to the guanylate cyclase domain, potentially jeopardizing the process that produces cytoplasmic cyclic guanosine monophosphate (cGMP) from GTP (Figure 2E).

Proband BAB8908 was born to Turkish kindred (HOU3212) with reported parental consanguinity, and the clinical phenotype was manifested by marked bilateral hallux macrodactyly with brachydactyly of the other toes (Figures 2A and 2B). Trio-ES analysis identified a homozygous missense variant allele (c.1673T>C [p.Ile558Thr]) in the kinase homology domain (KHD) of *NPR2* (Figures 2B–2D). Segregation analysis confirmed homozygosity for this variant allele in the proband; homozygosity was also found in an affected sister and affected maternal aunt of the proband. Both parents and the unaffected brother were heterozygous carriers consistent with Mendelian expectations (Figure 2A). The same allele has been reported previously in two affected siblings with short-limbed short stature in association with brachycephaly and a dysplastic middle phalanx of the fifth finger.⁴² AOH studies map the allelic variant to a 5.2 Mb AOH block, whereas the total AOH was 281.6 Mb, consistent with a clan genomics-derived IBD allele versus a Turkish IBS founder allele (Figure 2D).^{14,29,44}

WNT10B dysfunction in limb development and evidence of a potential founder allele

Failure of AER (Figure 1) stratification presumably results in split-hand/foot malformations (SHFM), a subgroup of CLM characterized by maldevelopment of central rays of the distal extremities. SHFM type 6 (SHFM6; MIM: 225300) is a non-syndromic AR trait associated with bial-

lelic frameshift, nonsense, or missense variants of *WNT10B*.^{45,46} Studies in mouse embryonic limb bud development reveal *Wnt10b* as an endogenous ectodermal Wnt ligand specifically expressed in the AER at embryonic day 11.5 (E11.5), whereas its regulatory mechanism remains largely unclear. In this cohort, we identified a novel indel variant that leads to a nonsense allele of *WNT10B* (c.741delC [p.Cys247*]); autozygosity of this novel frameshift allele causes a similar SHFM6 phenotype in two unrelated consanguineous Turkish families in our cohort because of IBD (Figures 3A and 3C).

In family HOU2346, two male siblings, BAB6262 and BAB8610, manifest an isolated bilateral split-foot malformation characterized by absence of the two central rays. In the upper distal limb, syndactyly is present between the third and fourth digit of the right hand of the proband (BAB6262), whereas his affected brother (BAB8610) has a flexion contracture of his left index finger (Figure 3B). The same *WNT10B* frameshift variant allele (c.741delC [p.Cys247*]) was identified in another consanguineous Turkish family in our cohort, HOU3360; homozygosity segregated with SHFM6 phenotypes among three generations (Figure 3C). Similar to the clinical features in family HOU2346, bilateral SHFM with more severe foot manifestations appeared among three affected individuals in family HOU3360 (proband BAB9281 and two siblings of her maternal grandfather, BAB9286 and BAB9316), in association with variable hand abnormalities, including syndactyly, polydactyly, and phalangeal dysplasia.

This frameshift variant allele (*WNT10B*, c.741delC) results in a PTC *in situ* (p.Cys247*) within the last exon; it is predicted by NMDescPredictor to escape NMD surveillance (Figure 3F). Compared with the *WNT10B* wild-type protein structure, the linker region and C-terminal domain (CTD) of *WNT10B* that is essential for formation of the Wnt-Frizzled-LRP6 ternary complex, is completely removed in the modeled *WNT10B* p.Cys247* truncation (Figure 3F). These observations suggest the underlying

(B) Clinical images and radiographs showing the hands and feet of two affected individuals (BAB6262 and BAB8610) in association with SHFM6. Note the right-hand 3-4 syndactyly in BAB6262 and hypoplasia with medial deviation of the index finger of the left hand of BAB6263. There is split foot malformation in both, with oligodactyly and syndactyly of lateral toes.

(C) Multigeneration pedigrees of family HOU3360, labeled with allelic information profiling segregation of the same frameshift variant (*WNT10B*: c.741delC [p.Cys247*]) and recessive inheritance pattern (i.e., AR trait) in comparison with family HOU2346. *BAB9287 is affected by an unknown type of CLM characterized by bilateral hand brachydactyly, clinodactyly, and phalangeal dysplasia. **BAB9284 has a unilateral polysyndactyly on the second toe of the right foot; its etiology remains unknown).

(D) Clinical photos of the three affected individuals in family HOU3360. From top to bottom: BAB9286, BAB9316, and BAB9281 manifest the SHFM6 phenotype, demonstrating varying degrees of SHFM, oligodactyly, and syndactyly.

(E) AOH genomic intervals mapping to chromosome 12 from ES personal genomes, visualized by B allele frequency computational studies from four affected individuals in families HOU2346 and HOU3360. The top two panels for family HOU2346 describe a 9.6 Mb (for BAB6262) and 21.6 Mb (for BAB8610) interval of AOH genomic interval haplotype block surrounding the causative variant allele of *WNT10B* (red vertical line), marked with thick gray rectangles. The two bottom panels for HOU3360 denote a shared 19.5 Mb AOH interval encompassing *WNT10B* variant alleles between individual BAB9281 (proband) and the affected brother of her maternal grandfather, BAB9286.

(F) An illustration from NMDescPredictor shows the *WNT10B* cDNA architecture and the position of the frameshift variant located in the NMD escape region (denoted as a green bar).

(G) The top illustration shows a comparison of linear structures or map positions and domains between the *WNT10B* wild-type (WT) and truncated version (p.Cys247*, with missing residues denoted by dashed lines). Also shown is a comparison (bottom) of two 3D protein structural models (*WNT10B*: WT and truncated version) obtained using the AlphaFold platform and visualized on UCSF Chimera (<https://www.cgl.ucsf.edu/chimera/>). Truncated *WNT10B* was predicted to lose the entire linker region and CTD, including the amino acids that map to the interface that interacts with the WNT classic receptor (Frizzled protein) and canonical coreceptor LRP5.

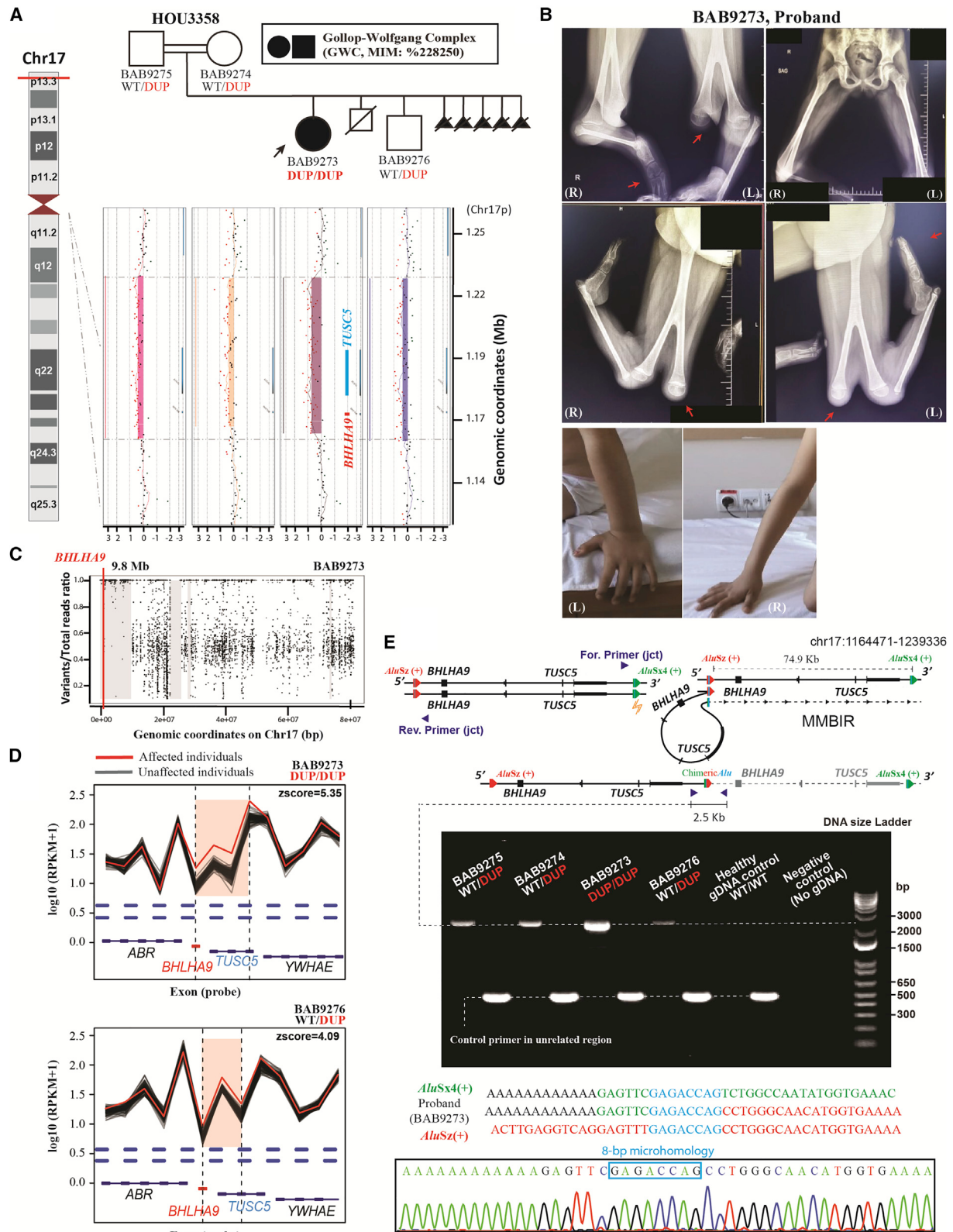

Figure 4. Homozygous duplication of *BHLHA9* causing GWC, illustrating semidominant inheritance

(A) Pedigree structure of HOU3358 with a customized HD-aCGH interrogating chr17p13.3 for each of the family members. An ~74.9 kb duplication encompassing the full-length of *BHLHA9* and *TUSC5* was identified in all family members. The proband (BAB9273) is homozygous for this duplication, and both parents (BAB9274 and BAB9275) and her brother (BAB9276) are unaffected heterozygous carriers.

(B) Clinical photos and radiographs of proband BAB9273, showing bifid femurs, tibial hemimelia, and monodactyly of the lower extremities; the upper extremities are observed as normotypical.

(legend continued on next page)

molecular mechanism and that the NMD-escaping frame-shift variant allele results in a truncated form of WNT10B that is unable to activate canonical Wnt/ β -catenin signaling pathways during AER stratification.

Segregation analysis and ROH/AOH profiling in these two multigeneration families revealed genomic evidence of consanguinity and autozygosity of disease-causing alleles (Figure 3E). In family HOU2346, the WNT10B variant maps within 9.6 Mb and 21.6 Mb genomic intervals of AOH/ROH in BAB6262 and BAB8610, with total AOH/ROH of 251.2 Mb and 217.2 Mb, respectively. In BAB9281 and BAB9286, a similar 19.5 Mb genomic interval of AOH/ROH encompasses WNT10B, which tracked with transmission of the disease-causing allele and provided evidence of intergeneration autozygosity through IBD. This genetic pattern, resulting from a homozygote marrying a recessive carrier, is referred to as a pseudodominant trait.

The youngest child, BAB6263 of family HOU2346, was found to have cerebral atrophy but no limb anomaly (Supplemental Note). Carrier and segregation analysis of BAB6263 identified a homozygous variant in general transcription factor IIIC subunit 1 (*GTF3C1*: c.4096G>A [p.Glu1366Lys]), and this particular *GTF3C1* variant allele has been reported previously by our group, causing cerebellar atrophy in another unrelated Turkish kindred (Figure S3).¹⁷ This family illustrates multi-locus pathogenic variation (MPV) within the personal genome of family members rather than the MPV of an individual with different genomic interval autozygosity in different siblings.

Haploinsufficiency of *HOXD* cluster deletion as a major molecular etiology for SHFM5

Large deletion CNVs encompassing the *HOXD* cluster have been proposed previously to cause syndromic SHFM5 (MIM: 606708), characterized by SHFM, syndactyly and skeletal hypoplasia in association with ectodermal and craniofacial findings.^{47–49}

We identified an individual (BAB4812) born to a Turkish family (HOU1397) with no report of parental consanguinity (Supplemental Note). His phenotype resembles a syndromic spectrum of SHFM5: bilateral hand SHFM and variable syndactyly; multiple congenital anomalies, including global developmental delay (GDD) and intellectual

disability (ID); hypotonia; and facial dysmorphism (Figures S4A and S4B).

CNV computational analysis from extant ES data of the BAB4812 personal genome via XHMM identified a *de novo*, ~7 Mb heterozygous deletion at chromosome 2q31.1. Subsequently, using customized HD-aCGH targeting and interrogating chromosome 2q, a *de novo* interstitial 7.8 Mb deletion, including 48 genes at chr2q31.1, was identified; this deletion CNV encompassed the entire *HOXD* gene cluster (*HOXD1-3-4-8-9-10-11-12-13*) and two adjacent regulatory topologically associating domains (TADs) (Figures S4A and S4C). Thus, the limb phenotype (SHFM5) of this individual is primarily derived from the haploinsufficiency of the entire *HOXD* territory.

BHLHA9 CNV and semidominant inheritance underlie GWC

Copy number gains of *BHLHA9* in humans have been described in association with the triplosensitivity trait (defined as a phenotype produced by an additional copy of the gene) potentially causing a gain-of-function (GoF) hypermorphic allele of SHFM with long bone deficiency (SHFLD3; MIM: 612576) as an AD trait with less than 50% penetrance and variable phenotypic expression.^{50–52} Family HOU3586 was referred from Brazil and has been published previously.⁵³ Three individuals manifest a paternally inherited CLM, including SHFM and bilateral tibial aplasia (Figures S5A–S5C). The whole-genome aCGH of each family member revealed a shared 61.8 kb duplication at chr17p13.3 in three affected and three unaffected individuals. The duplication encompasses *BHLHA9* driving the onset of SHFLD3.^{50–52}

GWC (MIM: 228250) is characterized by unilateral bifid femur and tibial bone absence with monodactyly.^{54,55} The presence of heterozygous triplication involving *BHLHA9* (gene copy number = 4) has been reported only once in a single family associated with GWC in the Japanese population, whereas there remains a lack of additional cases reported.⁵⁶ Here we found a novel allelic contribution by autozygosity-induced homozygous duplication of chr17p13.3, including the *BHLHA9* locus. HOU3358 is a consanguineous family from Syria in which proband BAB9273 was clinically diagnosed as having GWC

(C) B allele frequency of chromosome 17 for the proband showing a 9.8 Mb interval of AOH surrounding the homozygous *BHLHA9* duplication, marked with a thick gray line.

(D) A comparison of log10 reads per thousand base pairs per million reads (RPKM) plots of the duplication region from XHMM raw data. RPKM calculations of the proband (red line) and brother (yellow line) show different amplification levels on the reads mapping to the interrogated region (shaded in pink) in contrast to the gray lines delineated as individual ES samples from an internal database with similar experimental conditions.

(E) An illustration showing the proposed model of *BHLHA9* tandem duplication formed by AAMR. A single-strand break that occurred in the 3' *AluSx4+* element (yellow mark) introduced microhomology (marked by a blue bar)-mediated TS into another *AluSz+* element located in the 74.9 kb upstream region. A pair of primers was designed to capture the recombinant joint of the tandem duplicated allele by producing an amplicon size of ~2.5 kb encompassing the approximate breakpoint location. A control primer pair was applied on an ~500 bp unrelated genomic region. The diagram shown below is the 1% agarose electrophoresis of the PCR product; the specific band of ~2.5 kb is the tandem duplicated allele captured in all members (parents and brother, with the lighter bands denoting heterozygous duplication; while proband with a thick band consistent with homozygous duplication). Sanger sequencing (bottom) of the ~2.5 kb PCR product of the proband revealed an 8 bp microhomology. Three sequences above the Sanger sequencing trace from top to bottom denote the 3' *AluSx4+* reference (colored in green), the proband "chimeric" sequence, and the 5' *AluSz+* reference.

(Figures 4A and 4B). The proband has two unaffected brothers. The parents also had five pregnancies with fetuses that were prenatally diagnosed by ultrasound with bilateral femur bifurcations.

Initially, CNV analysis using XHMM from family-based exome data detected a potential copy number gain on chr17p13.3 involving *BHLHA9* in proband BAB9273 and her unaffected brother, BAB9276 (Figure 4D). Validation studies of each family member via customized chr17p HD-aCGH resolved a 74.9 kb duplication at chr17p13.3 covering the full length of *BHLHA9* and tumor suppressor candidate gene 5 (*TUCSS5*) in all four family members (Figure 4A). This duplicated allele was brought into homozygosity in the proband with GWC through IBD, not IBS, resulting in four genomic copies of the gene and genomic duplication interval (Figures 4A and 4C), a gene copy number identical to the heterozygous triplication mentioned above.⁵⁷

Semidominant inheritance, with a more severe phenotype in the homozygote versus heterozygote, indicates that the individuals who carried heterozygous variant alleles manifest an intermediate phenotype in comparison with the homozygous individual with the same duplication CNV affecting both alleles. Semidominant inheritance has been documented in other human disease traits, such as achondroplasia (ACH; point mutation of *FGFR3*; MIM: 100800) and Huntington disease (*HTT* triplet-repat polyglutamine expansion; MIM: 143100), and in gene duplication traits such as that resulting from *CMT1A* duplication.^{58,59} Here, homozygous duplication for *BHLHA9* results in GWC, a more severe type of limb malformation than SHFLD3; the latter is a rare disease trait caused by heterozygous duplication.

Structural variant (SV) mutagenesis by AAMR

To understand mutagenesis mechanisms underlying the observed CNV events, we applied breakpoint junction analysis on all SV cases, including deletion or duplication CNV alleles. For the *de novo* *HOXD* cluster deletion of proband BAB4812 in family HOU1397, analysis of this deleted CNV allele unveiled two flanking *Alu* elements (*AluSx+* and *AluSc8-*), in the opposite orientation with respect to the haploid reference genome, that generated a complex genomic rearrangement best described as a “deletion-normal/inversion-deletion (DEL-INV-DEL)” sequence involving a two-step template-switching (TS) mechanism (Figures S4D–S4F).⁶⁰ A 5 bp sequence microhomology and a 6 bp microhomology were found at two breakpoint junctions, respectively, indicating microhomology-mediated break-induced replication (MMBIR) as the likely underlying DNA-replicative repair mutational molecular mechanism (Figures S4D–S4F).

It has been established that chr17p13 is a “genomic instability hotspot” with high susceptibility for genomic rearrangement that frequently involves *Alu/Alu* recombination.⁶¹ *AluAluCNVPredictor* revealed that *BHLHA9* showed an elevated genomic instability index with a high relative risk score of 0.798 for OMIM genes and

0.782 for RefSeq genes (Figure S5E).²⁷ Breakpoint junction analysis of family HOU3586 showed a chimeric repetitive element fused by an *AluSx1* and a few upstream nucleotides adjacent to a long terminal repeat (LTR), potentially derived by MMBIR (Figure S5D). Strikingly, another duplicated allele, including *BHLHA9* in family HOU3358, was also demonstrated to be driven by an AAMR mechanism, resulting in a chimeric *Alu* element resulting from recombination of *AluSx4* and *AluSz* in the same orientation, with an 8 bp microhomology at the breakpoint junction (Figure 4E). Thus, *de novo* mutagenesis in an antecedent generation of the clan derived this allele by SV mutagenesis via AAMR, with IBD bringing it to homozygosity.

Discussion

Novel variant identification facilitating discovery of an allelic series

Family-based genomics studies and rare variant analyses in human populations throughout the world provide an opportunity to decipher a diverse and complex variant spectrum of disease trait-associated loci potentially contributing to CLM; such allelic diversity would not be sufficiently seen/or easily engineered in model organisms.^{14,44} Identifying complex variation (such as NMD-escaping variant alleles, gene triplication, etc.) provides new insights into the underlying mutational mechanisms enabling extensive allelic series and genotype-phenotype correlation analysis of a given disease-causing locus.

Allelic series is historically a genetic term referring to different “mutant alleles” of a gene or genomic locus that can generate a range of phenotypic trait manifestations. Each phenotype of this range is ascribed to variant alleles within different regions of the same gene/locus.⁶² For instance, *GLI3* and *HOXD13* are two genes regulating limb A/P axial development, and their disruption can cause AD-inherited digit abnormalities, as we showed in the families HOU2084 and HOU3022. However, allelic series in both genes are rather intriguing; extensive variant analysis on genotype-phenotype correlation of *GLI3* discovered a unique dichotomous relationship between the typical Greig cephalopolysyndactyly syndrome (GCPS) spectrum (MIM: 175700) and a more severe AD disorder called Pallister-Hall syndrome (PHS; MIM: 146510), depending on the location (i.e., genomic map position) of the causative variant allele.⁶³

In contrast to *GLI3*, similar observational studies of *HOXD13* revealed that its disruption was linked to many different digital malformations, including synpolydactyly (SPD1; MIM: 186000), brachydactyly type D (BDD; MIM: 113200), and brachydactyly type E (BDE; MIM: 113300). Although heterozygous polyalanine expansions, frame-shift, and nonsense variant alleles of *HOXD13* predominantly associate with SPD1, missense variants have been reported to cause SPD1 or BDD/BDE with no positional differentiation.

“Mirror trait” conditions are special allelic series that are defined as resulting from reciprocal genetic/genomic variants (e.g., duplication versus deletion CNV, LoF versus GoF) of the same gene/loci and can cause the observed phenotypes in individuals to appear at the opposite ends of a phenotypic spectrum (e.g., short stature versus tall stature).^{57,64} As we addressed previously, biallelic LoF variants of *NPR2* were frequently reported, causing AMDM characterized by short-limbed short stature. However, a few of the heterozygous missense variant and in-frame deletion alleles of *NPR2* were also reported to cause AD epiphyseal chondrodysplasia, Miura type (ECDM; MIM: 615923), characterized by tall stature, arachnodactyly, and long/broad halluces and long metatarsals because of a GoF (potentially a hypermorphic allele) that results in overactivity of guanylate cyclase (Figure S6).^{65–67} Genotype-phenotype correlation of these reciprocal traits highlighted their potential underlying etiologies related to abnormal CNP-induced cGMP production. In contrast to AMDM, ECDM-causing alleles were seldomly described (Figure S6).

WNT signaling perturbation affecting P/D axes of limb development

Wnt ligands associated with canonical and non-canonical signaling cascades are essential for limb and skeletal development, particularly required for AER formation and maintenance during P/D and D/V limb bud axial patterning.^{68–71} Perturbation of seven genes (*DVL1*, *DVL2*, *DVL3*, *FZD2*, *NXN*, *ROR2*, and *WNT5A*) acting on non-canonical WNT/planar cell polarity (PCP) signaling pathways are known causes of Robinow syndrome (RS), a congenital skeletal dysplasia with mesomelic limb shortening, distinct craniofacial findings enabling a recognizable pattern of human malformation, and other characteristics.^{72–74} In association with clinical and locus heterogeneity, limb shortening has been described as a common endophenotype in RS, and mesomelia (disproportionately short middle portions of the limb) is the most often observed consistent associated clinical finding in individuals with RS.^{74,75}

Although biallelic variants of *WNT10B* have been described to cause SHFM6 because of LoF alleles, heterozygous missense variant alleles of *WNT10B*, like *WNT10A*, can result in tooth agenesis, selective type 8 (STHAG8; MIM: 617073) with no distinct mutational location or nature.^{76,77} Homozygous mutations in *WLS*, encoding the Wnt ligand secretion mediator essential for secretion of all Wnt proteins, cause human syndromic structural defects with bilateral split foot and syndactyly.⁷⁸ For human WNT signaling, we also reported limb findings of bilateral foot oligodactyly and left-hand syndactyly in the individual with a *de novo*, likely damaging variant allele of Nemo-like kinase (*NLK*), an inhibitor of the WNT/ β -catenin signaling pathway (Figure S7; Supplemental Note).^{79,80} These clinical observations may not be readily synthesized nor mutational bases discovered and assessed

in most standard model organism approaches; thus, the pioneering organism *Homo sapiens* must be utilized for the allelic series.

CNV of *BHLHA9*, evidence of extension of an allele-specific gene dosage effect model

Gene dosage is a measurement of the number of copies of a gene in a species genome, whose alteration can simply be made by a CNV encompassing the given locus or gene-transcriptional processes, such as TAD disruptions, that may affect gene action or expression potentially related to a position effect. Rare CNVs are prominently associated with disease in humans and defined the entire field of genomic disorders.^{81–84}

Disease-causing CNVs are often large (hundreds of kb to Mb sizes), can include more than 1 gene, and are often significantly enriched for genes for transcription factors and/or signaling molecules contributing to developmental processes. Such genes may be subject to a gene dosage effect and may be dosage sensitive, and this aspect may serve as the major determinant of its pathogenicity resulting in haploinsufficient and triplosensitive traits and even mirror traits.⁵⁷ CNV studies of dosage-sensitive genes have contributed significantly to the progress of disease gene discovery and genomic instability/genome integrity mutagenesis; i.e., SV mutagenesis of the human genome.

BHLHA9 regulates apoptosis during mammalian autopod skeletogenesis and patterning.^{85,86} In the *Bhlha9* knockout mouse, interdigital programmed apoptosis is jeopardized, resulting in various severities of syndactyly of the fingers and hindlimbs/forelimbs.^{85,86} Abnormal expression patterns of Trp63 and other AER morphogens have been observed in *Bhlha9* knockout mice.⁸⁶ Functional depletion of *BHLHA9* has also been observed in individuals with biallelic missense variants involving the DNA-binding domain of *BHLHA9*, causing mesoaxial synostotic syndactyly with phalangeal reduction (MSSD; MIM: 609432).⁸⁷ Complex camptosynpolydactyly (CCSPD; MIM: 6097539), characterized by grossly malformed hands with digits arising from the dorsum of the hand and synpolydactyly, has also been reported in one Indian family.⁸⁸ The *BHLHA9* variant allele we describe here was homozygous, with the position of amino acid change mapping adjacent to the residue substitution observed in association with MSSD.

BHLHA9 is essential for interdigital apoptosis in central limb mesenchyme cells, and biallelic LoF variants ablate this regulatory ability. We hypothesized that copy number gains involving *BHLHA9* might enhance its regulatory potential; thus, the apoptosis activity may be overly or ectopically increased in central limb mesenchymal cells during limb development, causing SHFM with or without long bone deficiency. These phenomena are somewhat analogous to a “mirror trait”,⁶⁴ this, together with its strict spatiotemporal expression pattern, suggested *BHLHA9* as a dosage-sensitive gene. Further support for that contention comes from disrupted TAD findings in association

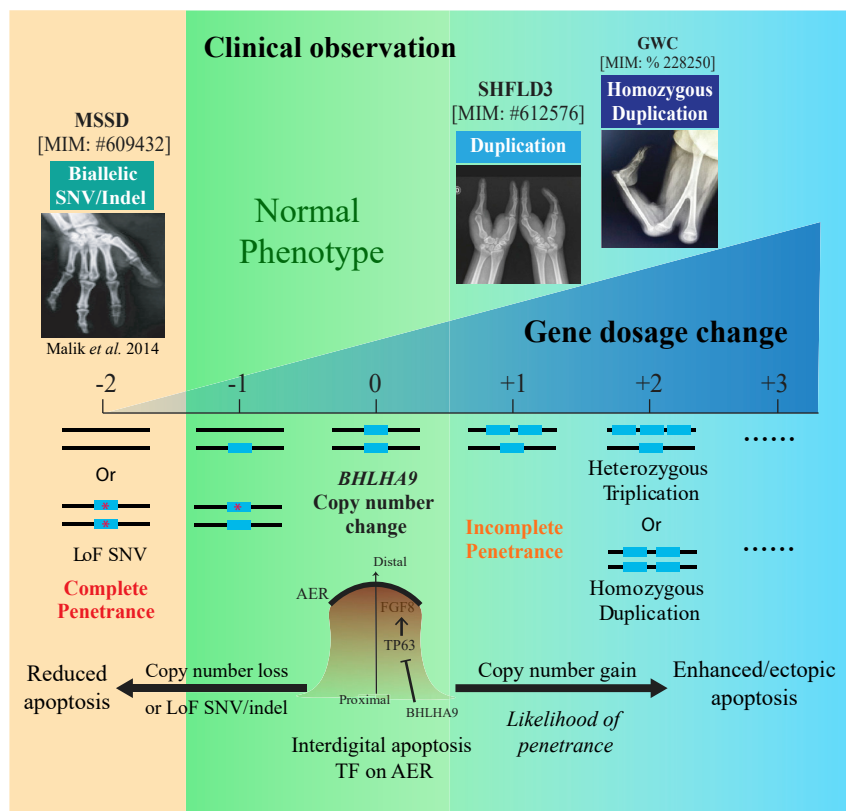

Figure 5. Allele-specific gene dosage model for perturbation of *BHLHA9* action

This illustration uses *BHLHA9* as a “molecular entry point” describing the potential genotype-phenotype correlation, penetrance, and expressivity. These observations can be parsimoniously explained by the perturbation of gene dosage underlying different genomic events, e.g., duplication versus triplication or quadruplication of the locus. Copy number gain of *BHLHA9* can abnormally enhance apoptosis during limb P/D development, causing the SHFLD phenotype. For individuals, an increased gene copy number increases the gene dosage, reinforcing the phenotype severity, likelihood, and penetrance, with homozygosity resulting in the most intense magnitude of effect (GWC). In contrast, biallelic LoF variant of *BHLHA9* result in an attenuated gene dosage, driving a distinct MSSD phenotype characterized by insufficient apoptosis activity during AER stratification.

Challenges and prospects

We provided further support for the contention that the etiological elements of developmental anomalies are rather heterogeneous. With current genome-wide methods (ES and aCGH in this study), we were incapable of

with duplication CNV.⁸⁹ Therefore, individually subtle differences of perturbations in gene expression caused by CNVs of *BHLHA9* may explain the phenotypic variability frequently described in the literature.^{90–92} Evidence shows that such dominant disease-causing CNVs with less than 50% penetrance, despite having a large effect size, could arise ancestrally and form as “founder pathogenic alleles” frequently transmitted in the Japanese population.⁵⁶ Mirror traits at the Smith-Magenis syndrome (MIM: 182290)/Potocki-Lupski syndrome (MIM: 610883) locus and the schizophrenia and autism chr1q21.1 loci associated with microcephaly/macrocephaly are mirror trait loci for loci manifesting gene dosage effects.^{57,64,93}

Triplication and quadruplication involving this locus have also been described in different families associated with SHFLD3 and GWC phenotypes in different populations, with the common observation that the copy number gain value (i.e., the number of gene copies gained) enhances the likelihood of penetrance and severity of the phenotype.^{56,88} We observed that homozygous duplication of *BHLHA9* can cause GWC, in which SHFLD3 can be seen as an intermediate phenotype. Here we propose that *BHLHA9*-associated limb malformations serve as a disease model with the association of penetrance and phenotype severity determined by its magnitude of effect on gene function (Figure 5).^{44,94–96} The resultant potential distortions of biological homeostasis could parsimoniously explain how such a “gene dosage expression model” significantly contributes to limb anomalies and other developmental birth defects.^{44,57}

revealing other types of genomic events, such as non-coding variant alleles (SNVs or CNVs), copy-number-neutral structural variants, and even some types of indels and repeat expansions (e.g., di-, tri-, and tetranucleotide repeats). Other factors known to cause limb defects, such as postzygotic/somatic mutations, mosaicism, fetal deformation, or exposure to environmental mutagens, are challenging to investigate and positively control.

Prospective short-read and long-read whole-genome sequencing (WGS), and RNA sequencing (RNA-seq) will be employed to detect additional causes in unsolved cases. Concerning overall phenotypic heterogeneity, human phenotype ontology (HPO)-based quantitative phenotypic analysis will be adapted in the future to quantify the limb anomaly trait and investigate the allele-specific differences (or to uncover novel allelic series) in similar CLM spectrums as well as to dissect MPV in AR or AD blended traits.^{74,97} Autozygosity mapping and AOH/ROH analyses from unphased exome data in consanguineous families could assist with additional novel disease gene/locus discovery in consanguineous families.⁹⁸ These strategies extend the effort to test the hypothesis proposed by Coban-Akdemir et al. that rare, deleterious variants embedded within long-sized AOH/ROH tend to evolve into disease-contributory haplotypes and whether the intrafamilial clinical heterogeneity could be the resultant from distributive AOH/ROH and MPV.³⁰ Interpretation of interconnections among allelic series, clinical severity, and reduced penetrance remains an important and challenging genotype/phenotype interpretive biology issue. Future

explorations will compare the allelic-specific gene dosage (AsGD) model proposed in this study with the established compound inheritance gene dosage model (CIGD) model to test this hypothesis and provide evidence as to whether such gene dosage expression models significantly contribute to limb anomalies and other developmental malformations.^{95,97}

Data and code availability

This study did not generate any codes or analyze any public datasets. The identified variants in this paper were submitted to the ClinVar with sequential identifiers SCV002546515–SCV002546524. All exome sequences, consent, and phenotypic data reported in this paper can be requested from controlled-access databases on dbGaP: phs000711.v7.p2.

Supplemental information

Supplemental information can be found online at <https://doi.org/10.1016/j.xhgg.2022.100132>.

Acknowledgments

We thank all families for participation in the study and dedicate this study to Prof. Temtamy and Prof. McKusick for their inspiration. This study was supported in part by the National Human Genome Research Institute (NHGRI) and National Heart, Lung, and Blood Institute (NHBLI) (to the Baylor-Hopkins Center for Mendelian Genomics [BHMG; UM1 HG006542] and Baylor College of Medicine Genomics Research to Elucidate the Genetics of Rare disease [BCM-GREGOR; U01 HG011758]), the National Institute of Neurological Disorders and Stroke (NINDS; R35NS105078 to J.R.L.), the Muscular Dystrophy Association (MDA; 512848 to J.R.L.), and the Spastic Paraplegia Foundation (SPF; to J.R.L.). J.E.P. was supported by NHGRI K08 HG008986. D.P. is supported by the International Rett Syndrome Foundation (IRSF; 3701-1).

Declaration of interests

J.R.L. has stock ownership in 23andMe, is a paid consultant for the Regeneron Genetics Center, and is a co-inventor on multiple United States and European patents related to molecular diagnostics for inherited neuropathies, eye diseases, genomic disorders, and bacterial genomic fingerprinting. The Department of Molecular and Human Genetics at Baylor College of Medicine receives revenue from clinical genetic testing and genomic testing (ES, WGS, CMA, and aCGH) conducted at Baylor Genetics (BG). J.R.L. serves on the Scientific Advisory Board (SAB) of BG.

Received: March 7, 2022

Accepted: July 19, 2022

Web Resources

1000 Genomes Project (TGP), <https://www.internationalgenome.org/>

Agilent aCGH for Genomic DNA Analysis with Tecan HS Pro Hybridization Station

https://www.agilent.com/cs/library/usermanuals/public/G4410-90011_CGH_Tecan_6.1.pdf

Agilent Genomic Workbench 7.0,

https://www.agilent.com/library/usermanuals/Public/G3800-90047_ProductOverview.pdf

Agilent SureDesign eArray system, <https://earray.chem.agilent.com/suredesign/>

AluAluCNVpredictor, <http://alualucnvpredictor.research.bcm.edu:3838/>

ARIC database, <http://www2.csc.unc.edu/aric/>

BaF Calculator Browser, <https://github.com/BCM-Lups-kilab/BaFCalculator>

BCM-HGSC Mercury analysis pipeline, <https://www.hgsc.bcm.edu/software/mercury>.

CADD, <https://cadd.gs.washington.edu/>

ClinVar, <https://www.ncbi.nlm.nih.gov/clinvar/>

ESP Exome Variant Server, <https://evs.gs.washington.edu/EVS/>

HMZDelFinder Browser, <https://github.com/BCM-Lups-kilab/HMZDelFinder>

Human Gene Mutation Database (HGMD), <http://www.hgmd.cf.ac.uk>

NMDescPredictor, <https://nmdprediction.shinyapps.io/nmdescpredictor/>

Online Mendelian Inheritance in Man, <https://www.omim.org/>

PubMed, <https://pubmed.ncbi.nlm.nih.gov/>

The Genome Aggregation Database (gnomAD), <https://gnomad.broadinstitute.org/>

UCSC Genome Browser, <https://genome.ucsc.edu/>

UCSF Chimera, <https://www.cgl.ucsf.edu/chimera/>

UniProt, <https://www.uniprot.org/>

XHMM, <http://atgu.mgh.harvard.edu/xhmm/index.shtml>

References

1. Zeller, R., López-Ríos, J., and Zuniga, A. (2009). Vertebrate limb bud development: moving towards integrative analysis of organogenesis. *Nat. Rev. Genet.* 10, 845–858. <https://doi.org/10.1038/nrg2681>.
2. Petit, F., Sears, K.E., and Ahituv, N. (2017). Limb development: a paradigm of gene regulation. *Nat. Rev. Genet.* 18, 245–258. <https://doi.org/10.1038/nrg.2016.167>.
3. Niswander, L. (2003). Pattern formation: old models out on a limb. *Nat. Rev. Genet.* 4, 133–143. <https://doi.org/10.1038/nrg1001>.
4. Biesecker, L.G., Aase, J.M., Clericuzio, C., Gurrieri, F., Temple, I.K., and Toriello, H. (2009). Elements of Morphology : Standard Terminology for the Hands and Feet. <https://doi.org/10.1002/ajmg.a.32596>.
5. Giele, H., Giele, C., Bower, C., and Allison, M. (2001). The incidence and epidemiology of congenital upper limb anomalies: a total population study. *J. Hand Surg. Am.* 26, 628–634. <https://doi.org/10.1053/jhsu.2001.26121>.
6. Ekblom, A.G., Laurell, T., and Arner, M. (2014). Epidemiology of congenital upper limb anomalies in stockholm, Sweden, 1997 to 2007: application of the oberg, manske, and tonkin

- classification. *J. Hand Surg. Am.* 39, 237–248. <https://doi.org/10.1016/j.jhsa.2013.11.014>.
7. Jourdain, A.S., Petit, F., Odou, M.F., Balduyck, M., Brunelle, P., Dufour, W., Boussion, S., Brischoux-Boucher, E., Colson, C., Dieux, A., et al. (2020). Multiplex targeted high-throughput sequencing in a series of 352 patients with congenital limb malformations. *Hum. Mutat.* 41, 222–239. <https://doi.org/10.1002/humu.23912>.
8. Sun, L., Huang, Y., Zhao, S., Zhao, J., Yan, Z., Guo, Y., Lin, M., Zhong, W., Yin, Y., Chen, Z., et al. (2021). Deciphering the mutational signature of congenital limb malformations. *Mol. Ther. Nucleic Acids* 24, 961–970. <https://doi.org/10.1016/j.omtn.2021.04.012>.
9. Elsner, J., Mensah, M.A., Holtgrewe, M., Hertzberg, J., Bigoni, S., Busche, A., Coutelier, M., de Silva, D.C., Elçioğlu, N., Filges, I., et al. (2021). Genome sequencing in families with congenital limb malformations. *Hum. Genet.* 140, 1229–1239. <https://doi.org/10.1007/s00439-021-02295-y>.
10. Yamoto, K., Saito, H., Nishimura, G., Kosaki, R., Takayama, S., Haga, N., Tonoki, H., Okumura, A., Horii, E., Okamoto, N., et al. (2019). Comprehensive clinical and molecular studies in split-hand/foot malformation: identification of two plausible candidate genes (*LRP6* and *UBA2*). *Eur. J. Hum. Genet.* 27, 1845–1857. <https://doi.org/10.1038/s41431-019-0473-7>.
11. Furniss, D., Kan, S.H., Taylor, I.B., Johnson, D., Critchley, P.S., Giele, H.P., and Wilkie, A.O.M. (2009). Genetic screening of 202 individuals with congenital limb malformations and requiring reconstructive surgery. *J. Med. Genet.* 46, 730–735. <https://doi.org/10.1136/jmg.2009.066027>.
12. Flöttmann, R., Kragesteen, B.K., Geuer, S., Socha, M., Allou, L., Sowińska-Seidler, A., Bosquillon de Jarcy, L., Wagner, J., Jamsheer, A., Oehl-Jaschkowitz, B., et al. (2018). Noncoding copy-number variations are associated with congenital limb malformation. *Genet. Med.* 20, 599–607. <https://doi.org/10.1038/gim.2017.154>.
13. Allou, L., Balzano, S., Magg, A., Quinodoz, M., Royer-Bertrand, B., Schöpflin, R., Chan, W.L., Speck-Martins, C.E., Carvalho, D.R., Farage, L., et al. (2021). Non-coding deletions identify Maenli lncRNA as a limb-specific *En1* regulator. *Nature* 592, 93–98. <https://doi.org/10.1038/s41586-021-03208-9>.
14. Lupski, J.R., Belmont, J.W., Boerwinkle, E., and Gibbs, R.A. (2011). Clan genomics and the complex architecture of human disease. *Cell* 147, 32–43. <https://doi.org/10.1016/j.cell.2011.09.008>.
15. Posey, J.E., O'Donnell-Luria, A.H., Chong, J.X., Harel, T., Jhangiani, S.N., Coban Akdemir, Z.H., Buyske, S., Pehlivan, D., Carvalho, C.M.B., Baxter, S., et al. (2019). Insights into genetics, human biology and disease gleaned from family based genomic studies. *Genet. Med.* 21, 798–812. <https://doi.org/10.1038/s41436-018-0408-7>.
16. Pehlivan, D., Bayram, Y., Gunes, N., Coban Akdemir, Z., Shukla, A., Bierhals, T., Tabakci, B., Sahin, Y., Gezdirici, A., Fatih, J.M., et al. (2019). The genomics of arthrogryposis, a complex trait: candidate genes and further evidence for oligogenic inheritance. *Am. J. Hum. Genet.* 105, 132–150. <https://doi.org/10.1016/j.ajhg.2019.05.015>.
17. Karaca, E., Harel, T., Pehlivan, D., Jhangiani, S.N., Gambin, T., Coban Akdemir, Z., Gonzaga-Jauregui, C., Erdin, S., Bayram, Y., Campbell, I.M., et al. (2015). Genes that affect brain structure and function identified by rare variant analyses of mendelian neurologic disease. *Neuron* 88, 499–513. <https://doi.org/10.1016/j.neuron.2015.09.048>.
18. Bainbridge, M.N., Wang, M., Wu, Y., Newsham, I., Muzny, D.M., Jefferies, J.L., Albert, T.J., Burgess, D.L., and Gibbs, R.A. (2011). Targeted enrichment beyond the consensus coding DNA sequence exome reveals exons with higher variant densities. *Genome Biol.* 12, R68. <https://doi.org/10.1186/gb-2011-12-7-r68>.
19. Reid, J.G., Carroll, A., Veeraraghavan, N., Dahdouli, M., Sundquist, A., English, A., Bainbridge, M., White, S., Salerno, W., Buhay, C., et al. (2014). Launching genomics into the cloud: deployment of Mercury, a next generation sequence analysis pipeline. *BMC Bioinf.* 15, 30. <https://doi.org/10.1186/1471-2105-15-30>.
20. Eldomery, M.K., Coban-Akdemir, Z., Harel, T., Rosenfeld, J.A., Gambin, T., Stray-Pedersen, A., Küry, S., Mercier, S., Lessel, D., Denecke, J., et al. (2017). Lessons learned from additional research analyses of unsolved clinical exome cases. *Genome Med.* 9, 26. <https://doi.org/10.1186/s13073-017-0412-6>.
21. Rentzsch, P., Witten, D., Cooper, G.M., Shendure, J., and Kircher, M. (2019). CADD: predicting the deleteriousness of variants throughout the human genome. *Nucleic Acids Res.* 47, D886–D894. <https://doi.org/10.1093/nar/gky1016>.
22. Ioannidis, N.M., Rothstein, J.H., Pejaver, V., Middha, S., McDonnell, S.K., Baheti, S., Musolf, A., Li, Q., Holzinger, E., Karyadi, D., et al. (2016). REVEL: an Ensemble method for predicting the pathogenicity of rare missense variants. *Am. J. Hum. Genet.* 99, 877–885. <https://doi.org/10.1016/j.ajhg.2016.08.016>.
23. Jarvik, G.P., and Browning, B.L. (2016). Consideration of cosegregation in the pathogenicity classification of genomic variants. *Am. J. Hum. Genet.* 98, 1077–1081. <https://doi.org/10.1016/j.ajhg.2016.04.003>.
24. Gambin, T., Akdemir, Z.C., Yuan, B., Gu, S., Chiang, T., Carvalho, C.M.B., Shaw, C., Jhangiani, S., Boone, P.M., Eldomery, M.K., et al. (2017). Homozygous and hemizygous CNV detection from exome sequencing data in a Mendelian disease cohort. *Nucleic Acids Res.* 45, 1633–1648. <https://doi.org/10.1093/nar/gkw1237>.
25. Fromer, M., Moran, J.L., Chambert, K., Banks, E., Bergen, S.E., Ruderfer, D.M., Handsaker, R.E., McCarroll, S.A., O'Donovan, M.C., Owen, M.J., et al. (2012). Discovery and statistical genotyping of copy-number variation from whole-exome sequencing depth. *Am. J. Hum. Genet.* 91, 597–607. <https://doi.org/10.1016/j.ajhg.2012.08.005>.
26. Pehlivan, D., Hullings, M., Carvalho, C.M.B., Gonzaga-Jauregui, C.G., Loy, E., Jackson, L.G., Krantz, I.D., Deardorff, M.A., and Lupski, J.R. (2012). *NIPBL* rearrangements in Cornelia de Lange syndrome: evidence for replicative mechanism and genotype-phenotype correlation. *Genet. Med.* 14, 313–322. <https://doi.org/10.1038/gim.2011.13>.
27. Song, X., Beck, C.R., Du, R., Campbell, I.M., Coban-Akdemir, Z., Gu, S., Breman, A.M., Stankiewicz, P., Ira, G., Shaw, C.A., and Lupski, J.R. (2018). Predicting human genes susceptible to genomic instability associated with *Alu/Alu*-mediated rearrangements. *Genome Res.* 28, 1228–1242. <https://doi.org/10.1101/gr.229401.117>.
28. Duan, R., Saadi, N.W., Grochowski, C.M., Bhadila, G., Farioun, A., Mitani, T., Du, H., Fatih, J.M., Jhangiani, S.N., Akdemir, Z.C., et al. (2021). A novel homozygous *SLC13A5* whole-gene deletion generated by *Alu/Alu*-mediated rearrangement in an Iraqi family with epileptic encephalopathy. *Am. J. Med. Genet.* 185, 1972–1980. <https://doi.org/10.1002/ajmg.a.62192>.

29. Mitani, T., Isikay, S., Gezdirici, A., Gulec, E.Y., Punetha, J., Fatihi, J.M., Herman, I., Akay, G., Du, H., Calame, D.G., et al. (2021). High prevalence of multilocus pathogenic variation in neurodevelopmental disorders in the Turkish population. *Am. J. Hum. Genet.* 108, 1981–2005. <https://doi.org/10.1016/j.ajhg.2021.08.009>.
30. Coban-Akdemir Z, Song X, Ceballos FC, et al. *De novo* mutation and identity-by-descent drive disease haplotypes, biallelic traits and multilocus pathogenic variation. *bioRxiv*. Published online July 27, 2022:2020.04.27.064824. <https://doi.org/10.1101/2020.04.27.064824>.
31. Karaca, E., Posey, J.E., Coban Akdemir, Z., Pehlivan, D., Harel, T., Jhangiani, S.N., Bayram, Y., Song, X., Bahrambeigi, V., Yuregir, O.O., et al. (2018). Phenotypic expansion illuminates multilocus pathogenic variation. *Genet. Med.* 20, 1528–1537. <https://doi.org/10.1038/gim.2018.33>.
32. Riggs, E.R., Andersen, E.F., Cherry, A.M., Kantarci, S., Kearney, H., Patel, A., Raca, G., Ritter, D.I., South, S.T., Thorland, E.C., et al. (2020). Technical standards for the interpretation and reporting of constitutional copy-number variants: a joint consensus recommendation of the American College of Medical Genetics and Genomics (ACMG) and the Clinical Genome Resource (ClinGen). *Genet. Med.* 22, 245–257. <https://doi.org/10.1038/s41436-019-0686-8>.
33. Richards, S., Aziz, N., Bale, S., Bick, D., Das, S., Gastier-Foster, J., Grody, W.W., Hegde, M., Lyon, E., Spector, E., et al. (2015). Standards and guidelines for the interpretation of sequence variants: a joint consensus recommendation of the American College of medical genetics and genomics and the association for molecular Pathology. *Genet. Med.* 17, 405–424. <https://doi.org/10.1038/gim.2015.30>.
34. Fujioka, H., Ariga, T., Horiuchi, K., Otsu, M., Igawa, H., Kawashima, K., Yamamoto, Y., Sugihara, T., and Sakiyama, Y. (2005). Molecular analysis of non-syndromic preaxial polydactyly: preaxial polydactyly type-IV and preaxial polydactyly type-I. *Clin. Genet.* 67, 429–433. <https://doi.org/10.1111/j.1399-0004.2005.00431.x>.
35. Radhakrishna, U., Bornholdt, D., Scott, H.S., Patel, U.C., Rossier, C., Engel, H., Bottani, A., Chandal, D., Blouin, J.L., Solanki, J.V., et al. (1999). The phenotypic spectrum of *GLI3* morphopathies includes autosomal dominant preaxial polydactyly type-IV and postaxial polydactyly type-A/B; no phenotype prediction from the position of *GLI3* mutations. *Am. J. Hum. Genet.* 65, 645–655. <https://doi.org/10.1086/302557>.
36. Salsi, V., Vigano, M.A., Cocchiarella, F., Mantovani, R., and Zappavigna, V. (2008). Hoxd13 binds in vivo and regulates the expression of genes acting in key pathways for early limb and skeletal patterning. *Dev. Biol.* 317, 497–507. <https://doi.org/10.1016/j.ydbio.2008.02.048>.
37. Muragaki, Y., Mundlos, S., Upton, J., and Olsen, B.R. (1996). Altered growth and branching patterns in synpolydactyly caused by mutations in *HOXD13*. *Science* 272, 548–551. <https://doi.org/10.1126/science.272.5261.548>.
38. Brison, N., Debeer, P., and Tylzanowski, P. (2014). Joining the Fingers : A *HOXD13* Story. 37-48. <https://doi.org/10.1002/dvdy.24037>.
39. Goodman, F., Giovannucci-Uzielli, M.L., Hall, C., Reardon, W., Winter, R., and Scambler, P. (1998). Deletions in *HOXD13* segregate with an identical, novel foot malformation in two unrelated families. *Am. J. Hum. Genet.* 63, 992–1000. <https://doi.org/10.1086/302070>.
40. Kurban, M., Wajid, M., Petukhova, L., Shimomura, Y., and Christiano, A.M. (2011). A nonsense mutation in the *HOXD13* gene underlies synpolydactyly with incomplete penetrance. *J. Hum. Genet.* 56, 701–706. <https://doi.org/10.1038/jhg.2011.84>.
41. Fantini, S., Vaccari, G., Brison, N., Debeer, P., Tylzanowski, P., and Zappavigna, V. (2009). A G220V substitution within the N-terminal transcription regulating domain of *HOXD13* causes a variant synpolydactyly phenotype. *Hum. Mol. Genet.* 18, 847–860. <https://doi.org/10.1093/hmg/ddn410>.
42. Bartels, C.F., Bükülmez, H., Padayatti, P., Rhee, D.K., van Ravenswaaij-Arts, C., Pauli, R.M., Mundlos, S., Chitayat, D., Shih, L.Y., Al-Gazali, L.I., et al. (2004). Mutations in the transmembrane natriuretic peptide receptor NPR-B impair skeletal growth and cause acromesomelic dysplasia, type Maroteaux. *Am. J. Hum. Genet.* 75, 27–34. <https://doi.org/10.1086/422013>.
43. Plachy, L., Dusatkova, P., Maratova, K., Petruzekova, L., Zemkova, D., Elblova, L., Kucerova, P., Toni, L., Kolouskova, S., Snajderova, M., et al. (2020). *NPR2* variants are frequent among children with familial short stature and respond well to growth hormone therapy. *J. Clin. Endocrinol. Metab.* 105. dgaa037–e752. <https://doi.org/10.1210/clinem/dgaa037>.
44. Lupski, J.R. (2021). Clan genomics: from OMIM phenotypic traits to genes and biology. *Am. J. Med. Genet.* 185, 3294–3313. <https://doi.org/10.1002/ajmg.a.62434>.
45. Blattner, A., Huber, A.R., and Röthlisberger, B. (2010). Homozygous nonsense mutation in *WNT10B* and sporadic split-hand/foot malformation (SHFM) with autosomal recessive inheritance. *Am. J. Med. Genet.* 152A, 2053–2056. <https://doi.org/10.1002/ajmg.a.33504>.
46. Kantaputra, P.N., Kapoor, S., Verma, P., Intachai, W., and Ketudat Cairns, J.R. (2018). Split hand-foot malformation and a novel *WNT10B* mutation. *Eur. J. Med. Genet.* 61, 372–375. <https://doi.org/10.1016/j.ejmg.2018.02.001>.
47. Theisen, A., Rosenfeld, J.A., Shane, K., McBride, K.L., Atkin, J.F., Gaba, C., Hoo, J., Kurczynski, T.W., Schnur, R.E., Coffey, L.B., et al. (2010). Refinement of the region for split hand/foot malformation 5 on 2q31.1. *Mol. Syndromol.* 1, 262–271. <https://doi.org/10.1159/000328405>.
48. Del Campo, M., Jones, M.C., Veraksa, A.N., Curry, C.J., Jones, K.L., Mascarello, J.T., Ali-Kahn-Catts, Z., Drumheller, T., and McGinnis, W. (1999). Monodactylous limbs and abnormal genitalia are associated with hemizygosity for the human 2q31 region that includes the *HOXD* cluster. *Am. J. Hum. Genet.* 65, 104–110. <https://doi.org/10.1086/302467>.
49. Svensson, A.M., Curry, C.J., South, S.T., Whitby, H., Maxwell, T.M., Aston, E., Fisher, J., Carmack, C.E., Scheffer, A., Abu-Shamsieh, A., and Brothman, A.R. (2007). Detection of a *de novo* interstitial 2q microdeletion by CGH microarray analysis in a patient with limb malformations, microcephaly and mental retardation. *Am. J. Med. Genet.* 143, 1348–1353. <https://doi.org/10.1002/ajmg.a.31775>.
50. Petit, F., Jourdain, A.S., Andrieux, J., Baujat, G., Baumann, C., Beneteau, C., David, A., Faivre, L., Gaillard, D., Gilbert-Dussardier, B., et al. (2014). Split hand/foot malformation with long-bone deficiency and *BHLHA9* duplication: report of 13 new families. *Clin. Genet.* 85, 464–469. <https://doi.org/10.1111/cge.12219>.
51. Paththinige, C.S., Sirisena, N.D., Escande, F., Manouvrier, S., Petit, F., and Dissanayake, V.H.W. (2019). Split hand/foot malformation with long bone deficiency associated with *BHLHA9* gene duplication: a case report and review of literature. *BMC*

- Med. Genet. 20, 108–117. <https://doi.org/10.1186/s12881-019-0839-2>.
52. Klopocki, E., Lohan, S., Doelken, S.C., Stricker, S., Ockeloen, C.W., Soares Thiele de Aguiar, R., Lezirovitz, K., Mingroni Netto, R.C., Jamsheer, A., Shah, H., et al. (2012). Duplications of *BHLHA9* are associated with ectrodactyly and tibia hemimelia inherited in non-Mendelian fashion. *J. Med. Genet.* 49, 119–125. <https://doi.org/10.1136/jmedgenet-2011-100409>.
  53. da Rocha, L.A., Pires, L.V.L., Yamamoto, G.L., Magliocco Ceroni, J.R., Honjo, R.S., de Novaes França Bisneto, E., Oliveira, L.A.N., Rosenberg, C., Krepischi, A.C.V., Passos-Bueno, M.R., et al. (2021). Congenital limb deficiency: genetic investigation of 44 individuals presenting mainly longitudinal defects in isolated or syndromic forms. *Clin. Genet.* 100, 615–623. <https://doi.org/10.1111/cge.14041>.
  54. Wolfgang, G.L. (1984). Complex congenital anomalies of the lower extremities: femoral bifurcation, tibial hemimelia, and diastasis of the ankle. Case report and review of the literature. *J. Bone Joint Surg. Am.* 66, 453–458. https://journals.lww.com/jbjsjournal/Fulltext/1984/66030/Complex_congenital_anomalies_of_the_lower.21.aspx.
  55. Gollop, T.R., Lucchesi, E., Martins, R.M., Nione, A.S., and Optiz, J.M. (1980). Familial occurrence of bifid femur and monodactylous ectrodactyly. *Am. J. Med. Genet.* 7, 319–322. <https://doi.org/10.1002/ajmg.1320070313>.
  56. Nagata, E., Kano, H., Kato, F., Yamaguchi, R., Nakashima, S., Takayama, S., Kosaki, R., Tonoki, H., Mizuno, S., Watanabe, S., et al. (2014). Japanese founder duplications/triplications involving *BHLHA9* are associated with split-hand/foot malformation with or without long bone deficiency and Gollop-Wolfgang complex. *Orphanet J. Rare Dis.* 9, 125. <https://doi.org/10.1186/s13023-014-0125-5>.
  57. Lupski, J.R. (2022). Biology in balance: human diploid genome integrity, gene dosage, and genomic medicine. *Trends Genet.* 38, 554–571. <https://doi.org/10.1016/j.tig.2022.03.001>.
  58. Kaku, D.A., Parry, G.J., Malamut, R., Lupski, J.R., and Garcia, C.A. (1993). Nerve conduction studies in Charcot-Marie-Tooth polyneuropathy associated with a segmental duplication of chromosome 17. *Neurology* 43, 1806–1808. <https://doi.org/10.1212/WNL.43.9.1806>.
  59. Lupski, J.R., de Oca-Luna, R.M., Slaugenhaupt, S., Pentao, L., Guzzetta, V., Trask, B.J., Saucedo-Cardenas, O., Barker, D.F., Killian, J.M., Garcia, C.A., et al. (1991). DNA duplication associated with Charcot-Marie-Tooth disease type 1A. *Cell* 66, 219–232. [https://doi.org/10.1016/0092-8674\(91\)90613-4](https://doi.org/10.1016/0092-8674(91)90613-4).
  60. Carvalho, C.M.B., and Lupski, J.R. (2016). Mechanisms underlying structural variant formation in genomic disorders. *Nat. Rev. Genet.* 17, 224–238. <https://doi.org/10.1038/nrg.2015.25>.
  61. Gu, S., Yuan, B., Campbell, I.M., Beck, C.R., Carvalho, C.M.B., Nagamani, S.C.S., Erez, A., Patel, A., Bacino, C.A., Shaw, C.A., et al. (2015). *Alu*-mediated diverse and complex pathogenic copy-number variants within human chromosome 17 at p13.3. *Hum. Mol. Genet.* 24, 4061–4077. <https://doi.org/10.1093/hmg/ddv146>.
  62. (2006). Allelic Series. In *Encyclopedic Reference of Genomics and Proteomics in Molecular Medicine* Encyclopedic Reference of Genomics and Proteomics in Molecular Medicine (Springer Berlin Heidelberg), p. 43. https://doi.org/10.1007/3-540-29623-9_6099.
  63. Johnston, J.J., Olivos-Glander, I., Killoran, C., Elson, E., Turner, J.T., Peters, K.F., Abbott, M.H., Aughton, D.J., Aylsworth, A.S., Bamshad, M.J., et al. (2005). Molecular and clinical analyses of Greig cephalopolysyndactyly and pallister-hall syndromes: robust phenotype prediction from the type and position of *GLI3* mutations. *Am. J. Hum. Genet.* 76, 609–622. <https://doi.org/10.1086/429346>.
  64. Lupski, J.R. (2015). Structural variation mutagenesis of the human genome: impact on disease and evolution. *Environ. Mol. Mutagen.* 56, 419–436. <https://doi.org/10.1002/em.21943>.
  65. Hannema, S.E., van Duyvenvoorde, H.A., Premisler, T., Yang, R.B., Mueller, T.D., Gassner, B., Oberwinkler, H., Roelfsema, F., Santen, G.W.E., Prickett, T., et al. (2013). An activating mutation in the kinase homology domain of the natriuretic peptide receptor-2 causes extremely tall stature without skeletal deformities. *J. Clin. Endocrinol. Metab.* 98, E1988–E1998. <https://doi.org/10.1210/jc.2013-2358>.
  66. Miura, K., Kim, O.-H., Lee, H.R., Namba, N., Michigami, T., Yoo, W.J., Choi, I.H., Ozono, K., and Cho, T.J. (2014). Overgrowth syndrome associated with a gain-of-function mutation of the natriuretic peptide receptor 2 (*NPR2*) gene. *Am. J. Med. Genet.* 164A, 156–163. <https://doi.org/10.1002/ajmg.a.36218>.
  67. Lauffer, P., Miranda-Laferte, E., van Duyvenvoorde, H.A., van Haeringen, A., Werner, F., Boudin, E., Schmidt, H., Mueller, T.D., Kuhn, M., and van der Kaay, D.C.M. (2020). An activating deletion variant in the submembrane region of natriuretic peptide receptor-B causes tall stature. *J. Clin. Endocrinol. Metab.* 105, dgaa190-2366. <https://doi.org/10.1210/clinem/dgaa190>.
  68. Church, V.L., and Francis-West, P. (2002). Wnt signalling during limb development. *Int. J. Dev. Biol.* 46, 927–936. <https://doi.org/10.1387/ijdb.12455630>.
  69. Geetha-Loganathan, P., Nimmagadda, S., and Scaal, M. (2008). Wnt signaling in limb organogenesis. *Organogenesis* 4, 109–115. <https://doi.org/10.4161/org.4.2.5857>.
  70. Kawakami, Y., Capdevila, J., Büscher, D., Itoh, T., Rodríguez Esteban, C., and Izpisua Belmonte, J.C. (2001). WNT signals control FGF-dependent limb initiation and AER induction in the chick embryo. *Cell* 104, 891–900. [https://doi.org/10.1016/S0092-8674\(01\)00285-9](https://doi.org/10.1016/S0092-8674(01)00285-9).
  71. Witte, F., Dokas, J., Neuendorf, F., Mundlos, S., and Stricker, S. (2009). Comprehensive expression analysis of all *Wnt* genes and their major secreted antagonists during mouse limb development and cartilage differentiation. *Gene Expr. Patterns* 9, 215–223. <https://doi.org/10.1016/j.gep.2008.12.009>.
  72. Zhang, C., Mazzeu, J.F., Eisfeldt, J., Grochowski, C.M., White, J., Akdemir, Z.C., Jhangiani, S.N., Muzny, D.M., Gibbs, R.A., Lindstrand, A., et al. (2021). Novel pathogenic genomic variants leading to autosomal dominant and recessive Robinow syndrome. *Am. J. Med. Genet.* 185, 3593–3600. <https://doi.org/10.1002/ajmg.a.61908>.
  73. White, J.J., Mazzeu, J.F., Coban-Akdemir, Z., Bayram, Y., Bahrambeigi, V., Hoischen, A., van Bon, B.W.M., Gezdirici, A., Gullec, E.Y., Ramond, F., et al. (2018). WNT signaling perturbations underlie the genetic heterogeneity of Robinow syndrome. *Am. J. Hum. Genet.* 102, 27–43. <https://doi.org/10.1016/j.ajhg.2017.10.002>.
  74. Zhang, C., Jolly, A., Shayota, B.J., Mazzeu, J.F., Du, H., Dawood, M., Soper, P.C., Ramalho de Lima, A., Ferreira, B.M., Coban-Akdemir, Z., et al. (2022). Novel pathogenic variants and quantitative phenotypic analyses of Robinow syndrome: WNT signaling perturbation and phenotypic variability. *HGG Adv.* 3, 100074. <https://doi.org/10.1016/j.xhgg.2021.100074>.

75. Abu-Ghname, A., Trost, J., Davis, M.J., Sutton, V.R., Zhang, C., Guillen, D.E., Carvalho, C.M.B., and Maricevich, R.S. (2021). Extremity anomalies associated with Robinow syndrome. *Am. J. Med. Genet.* 185, 3584–3592. <https://doi.org/10.1002/ajmg.a.61884>.
76. Yu, P., Yang, W., Han, D., Wang, X., Guo, S., Li, J., Li, F., Zhang, X., Wong, S.W., Bai, B., et al. (2016). Mutations in *WNT10B* are identified in individuals with oligodontia. *Am. J. Hum. Genet.* 99, 195–201. <https://doi.org/10.1016/j.ajhg.2016.05.012>.
77. Du, R., Dinckan, N., Song, X., Coban-Akdemir, Z., Jhangiani, S.N., Guven, Y., Aktoren, O., Kayserili, H., Petty, L.E., Muzny, D.M., et al. (2018). Identification of likely pathogenic and known variants in *TSPEAR*, *LAMB3*, *BCOR*, and *WNT10A* in four Turkish families with tooth agenesis. *Hum. Genet.* 137, 689–703. <https://doi.org/10.1007/s00439-018-1907-y>.
78. Chai, G., Szenker-Ravi, E., Chung, C., Li, Z., Wang, L., Khatoo, M., Marshall, T., Jiang, N., Yang, X., McEvoy-Venneri, J., et al. (2021). A human pleiotropic multiorgan condition caused by deficient Wnt secretion. *N. Engl. J. Med.* 385, 1292–1301. <https://doi.org/10.1056/NEJMoa2033911>.
79. Ishitani, T., Ninomiya-Tsuji, J., and Matsumoto, K. (2003). Regulation of lymphoid enhancer factor 1/T-cell factor by mitogen-activated protein kinase-related Nemo-like kinase-dependent phosphorylation in Wnt/beta-catenin signaling. *Mol. Cell Biol.* 23, 1379–1389. <https://doi.org/10.1128/MCB.23.4.1379-1389.2003>.
80. Sokol, S.Y. (2011). Wnt signaling through T-cell factor phosphorylation. *Cell Res.* 21, 1002–1012. <https://doi.org/10.1038/cr.2011.86>.
81. Lupski, J.R., Wise, C.A., Kuwano, A., Pentao, L., Parke, J.T., Glaze, D.G., Ledbetter, D.H., Greenberg, F., and Patel, P.I. (1992). Gene dosage is a mechanism for Charcot-Marie-Tooth disease type 1A. *Nat. Genet.* 1, 29–33. <https://doi.org/10.1038/ng0492-29>.
82. Lupski, J.R. (1998). Genomic disorders: structural features of the genome can lead to DNA rearrangements and human disease traits. *Trends Genet.* 14, 417–422. [https://doi.org/10.1016/S0168-9525\(98\)01555-8](https://doi.org/10.1016/S0168-9525(98)01555-8).
83. Lupski, J.R. (2009). Genomic disorders ten years on. *Genome Med.* 1, 42–51. <https://doi.org/10.1186/gm42>.
84. Harel, T., and Lupski, J.R. (2018). Genomic disorders 20 years on—mechanisms for clinical manifestations. *Clin. Genet.* 93, 439–449. <https://doi.org/10.1111/cge.13146>.
85. Schatz, O., Langer, E., and Ben-Arie, N. (2014). Gene dosage of the transcription factor *Fingerin* (*bHLHA9*) affects digit development and links syndactyly to ectrodactyly. *Hum. Mol. Genet.* 23, 5394–5401. <https://doi.org/10.1093/hmg/ddu257>.
86. Kataoka, K., Matsushima, T., Ito, Y., Sato, T., Yokoyama, S., and Asahara, H. (2018). *Bhlha9* regulates apical ectodermal ridge formation during limb development. *J. Bone Miner. Metabol.* 36, 64–72. <https://doi.org/10.1007/s00774-017-0820-0>.
87. Malik, S., Percin, F.E., Bornholdt, D., Albrecht, B., Percesepe, A., Koch, M.C., Landi, A., Fritz, B., Khan, R., Mumtaz, S., et al. (2014). Mutations affecting the BHLHA9 DNA-binding domain cause MSSD, mesoaxial synostotic syndactyly with phalangeal reduction, malik-percin type. *Am. J. Hum. Genet.* 95, 649–659. <https://doi.org/10.1016/j.ajhg.2014.10.012>.
88. Phadke, S.R., Kar, A., Bhowmik, A.D., and Dalal, A. (2016). Complex Camptosynpolydactyly and Mesoaxial synostotic syndactyly with phalangeal reduction are allelic disorders. *Am. J. Med. Genet.* 170, 1622–1625. <https://doi.org/10.1002/ajmg.a.37643>.
89. Lupiáñez, D.G., Kraft, K., Heinrich, V., Krawitz, P., Brancati, F., Klopocki, E., Horn, D., Kayserili, H., Opitz, J.M., Laxova, R., et al. (2015). Disruptions of topological chromatin domains cause pathogenic rewiring of gene-enhancer interactions. *Cell* 161, 1012–1025. <https://doi.org/10.1016/j.cell.2015.04.004>.
90. Bien-Willner, G.A., Stankiewicz, P., and Lupski, J.R. (2007). SOX9cre1, a cis-acting regulatory element located 1.1 Mb upstream of *SOX9*, mediates its enhancement through the SHH pathway. *Hum. Mol. Genet.* 16, 1143–1156. <https://doi.org/10.1093/hmg/ddm061>.
91. Velagaleti, G.V.N., Bien-Willner, G.A., Northup, J.K., Lockhart, L.H., Hawkins, J.C., Jalal, S.M., Withers, M., Lupski, J.R., and Stankiewicz, P. (2005). Position effects due to chromosome breakpoints that map ~900 Kb upstream and ~1.3 Mb downstream of *SOX9* in two patients with campomelic dysplasia. *Am. J. Hum. Genet.* 76, 652–662. <https://doi.org/10.1086/429252>.
92. Zhang, F., Seeman, P., Liu, P., Weterman, M.A.J., Gonzaga-Jaurégui, C., Towne, C.F., Batish, S.D., De Vriendt, E., De Jonghe, P., Rautenstrauss, B., et al. (2010). Mechanisms for nonrecurrent genomic rearrangements associated with CMT1A or HNPP: rare CNVs as a cause for missing heritability. *Am. J. Hum. Genet.* 86, 892–903. <https://doi.org/10.1016/j.ajhg.2010.05.001>.
93. Lupski, J.R. (2008). Schizophrenia: incriminating genomic evidence. *Nature* 455, 178–179. <https://doi.org/10.1038/455178a>.
94. Wu, N., Ming, X., Xiao, J., Wu, Z., Chen, X., Shinawi, M., Shen, Y., Yu, G., Liu, J., Xie, H., et al. (2015). *TBX6* null variants and a common hypomorphic allele in congenital scoliosis. *N. Engl. J. Med.* 372, 341–350. <https://doi.org/10.1056/NEJMoa1406829>.
95. Liu, J., Wu, N., Deciphering Disorders Involving Scoliosis and COMorbidities DISCO study, Takeda, K., Chen, W., Li, W., Du, R., Liu, S., Zhou, Y., Zhang, L., et al. (2019). *TBX6*-associated congenital scoliosis (TACS) as a clinically distinguishable subtype of congenital scoliosis: further evidence supporting the compound inheritance and *TBX6* gene dosage model. *Genet. Med.* 21, 1548–1558. <https://doi.org/10.1038/s41436-018-0377-x>.
96. Chen, W., Lin, J., Wang, L., Li, X., Zhao, S., Liu, J., Akdemir, Z.C., Zhao, Y., Du, R., Ye, Y., et al. (2020). *TBX6* missense variants expand the mutational spectrum in a non-Mendelian inheritance disease. *Hum. Mutat.* 41, 182–195. <https://doi.org/10.1002/humu.23907>.
97. Herman, I., Jolly, A., Du, H., Dawood, M., Abdel-Salam, G.M.H., Marafi, D., Mitani, T., Calame, D.G., Coban-Akdemir, Z., Fatih, J.M., et al. (2022). Quantitative dissection of multilocus pathogenic variation in an Egyptian infant with severe neurodevelopmental disorder resulting from multiple molecular diagnoses. *Am. J. Med. Genet.* 188, 735–750. <https://doi.org/10.1002/ajmg.a.62565>.
98. Carr, I.M., Bhaskar, S., O'Sullivan, J., Aldahmesh, M.A., Shamseldin, H.E., Markham, A.F., Bonthron, D.T., Black, G., and Alkuraya, F.S. (2013). Autozygosity mapping with exome sequence data. *Hum. Mutat.* 34, 50–56. <https://doi.org/10.1002/humu.22220>.
99. Sezer, O., Gebesoglu, I., Yuan, B., Karaca, E., Gokce, E., and Gunes, S. (2014). Fibular aplasia, tibial campomelia, and oligosyndactyly: a further patient with a 2-year follow-up. *Clin. Dysmorphol.* 23, 121–126. https://journals.lww.com/clindysmorphol/Fulltext/2014/10000/Fibular_aplasia,_tibial_campomelia,_and.2.aspx.

Supplemental information

Developmental genomics of limb malformations:

Allelic series in association with gene

dosage effects contribute to the clinical variability

Ruizhi Duan, Hadia Hijazi, Elif Yilmaz Gulec, Hatice Koçak Eker, Silvia R. Costa, Yavuz Sahin, Zeynep Ocak, Sedat Isikay, Ozge Ozalp, Sevcin Bozdogan, Huseyin Aslan, Nursel Elcioglu, Débora R. Bertola, Alper Gezdirici, Haowei Du, Jawid M. Fatih, Christopher M. Grochowski, Gulsen Akay, Baylor-Hopkins Center for Mendelian Genomics, Shalini N. Jhangiani, Ender Karaca, Shen Gu, Zeynep Coban-Akdemir, Jennifer E. Posey, Yavuz Bayram, V. Reid Sutton, Claudia M.B. Carvalho, Davut Pehlivan, Richard A. Gibbs, and James R. Lupski

Table of Contents

**SUPPLEMENTAL NOTE: CASE REPORTS**	**2**
BAB4812	2
BAB6263	3
BAB11205	3
**SUPPLEMENTAL FIGURES**	**5**
Figure S1	5
Figure S2.....	7
Figure S3.....	8
Figure S4.....	10
Figure S5.....	13
Figure S6.....	15
Figure S7.....	16
**SUPPLEMENTAL MATERIAL AND METHODS**	**17**
PCR Primers used in this study	17

SUPPLEMENTAL NOTE: CASE REPORTS

BAB4812

BAB4812 is a male, born via C-section at 34 weeks of gestation per ultrasound measurements (38 4/7 per last menstrual period), with a birth weight of 1.88 kg (10-25%ile per 34 GW measurements). His parents were from the same Turkish village with no report of consanguinity. The index subject has a brother who was affected by hydrocephalus and died at 7-months of age, and the parents later had a healthy girl from their second pregnancy. The index subject was their third child. By physical examination at seven days of life, the subject weighed 2 kg (3-10%ile per 35 GW measurements) and measured 31.5 cm (10-50%ile per 35 GW measurements) in length. He had hypotonia, down-slanting palpebral fissures, micrognathia, *pectus carinatum*, bilateral split-hand malformation, bilateral syndactyly involving the 3rd and 4th toes, and 'paleness' on the inner sides of the legs (**Figure S4**). Laryngomalacia and unilateral vocal cord paralysis were identified by the ear nose & throat examination. The echocardiogram on the proband showed systolic dysfunction and valve insufficiency, and a troponin I was mildly elevated at 0.07 ng/ml (normal < 0.04 ng/ml) and attributed to perinatal asphyxia. Ophthalmologic evaluation was remarkable for bilateral intraretinal hemorrhages and lack of vascularization in the periphery of zone 3. Karyotype was normal, 46,XY. During the evaluation at 2-months of age, the index subject was hypotonic with a poor suck reflex. Anthropometric measurements revealed 2.8 kg (3%ile) weight and 34 cm (-7.3 *SD*) length. The proband was diagnosed with intellectual disability at 7 years old with delays in all developmental parameters. The most recent clinical evaluation at 8.5-years revealed measurements of 15 kg (-3.7 *SD*) weight and 102 cm (-4.9 *SD*) height. He was unable to speak or swallow, and had vomiting

(likely secondary to reflux). He had no appetite, could not eat solid food and was fed with formula. He had constipation without alternating diarrhea episodes. Laboratory tests including complete blood count, liver function tests, and thyroid function tests yielded unremarkable values. The celiac disease antibody test was negative.

BAB6263

BAB6263 was an 11-year-old male who developed seizures at 3-months of age. His parents were 1st cousins from Turkey. The subject displayed intellectual disability (ID) during the latest examination, and his brain MRI showed mild cerebellar atrophy (**Figure S3**). His renal ultrasound was normal. No additional abnormalities were identified in limb radiographs. He has two healthy sisters and two brothers (BAB6262 and BAB8610) who were only affected with split-hand/foot malformation and had normal cognition.

BAB11205

BAB11205 was a male subject and the twin product of *in vitro* fertilization. His twin resulted in miscarriage during pregnancy (**Figure S7**). His parents were from nearby villages with no report of known consanguinity. His parents reported three previous miscarriages. Prenatal ultrasound revealed short long bones and an increased nuchal fold. At 7-months of age, physical examination showed his head circumference to be 39 cm (-4 *SD*), weight 4.75 kg (-4.2 *SD*), and length 58 cm (-4.3 *SD*). At the 16-months of age evaluation, anthropometric measurements were: head circumference 42 cm (-4.1 *SD*), weight 7.4 kg (-3.5 *SD*) and height 70 cm (-3.3 *SD*). Physical examination was remarkable for microcephaly, open anterior fontanelle, narrow forehead, blepharophimosis, ptosis, thin lips, long fingers with partial syndactyly of left hand, bilateral single transverse palmar crease, short and bowed femurs, bilateral cryptorchidism, and

bilateral oligodactyly of the feet (**Figure S7**). Abdominal ultrasound showed left renal agenesis. Echocardiogram revealed multiple cardiac anomalies, including atrial septal defect, ventricular septal defect, peripheral pulmonary stenosis and mitral valve prolapse. No abnormal findings were noted on his electroencephalogram (EEG). His karyotype was normal 46, XY. Cranial MRI showed mild triventricular hydrocephalus. A 60K array CGH did not reveal any copy number variations.

SUPPLEMENTAL FIGURES

Figure S1

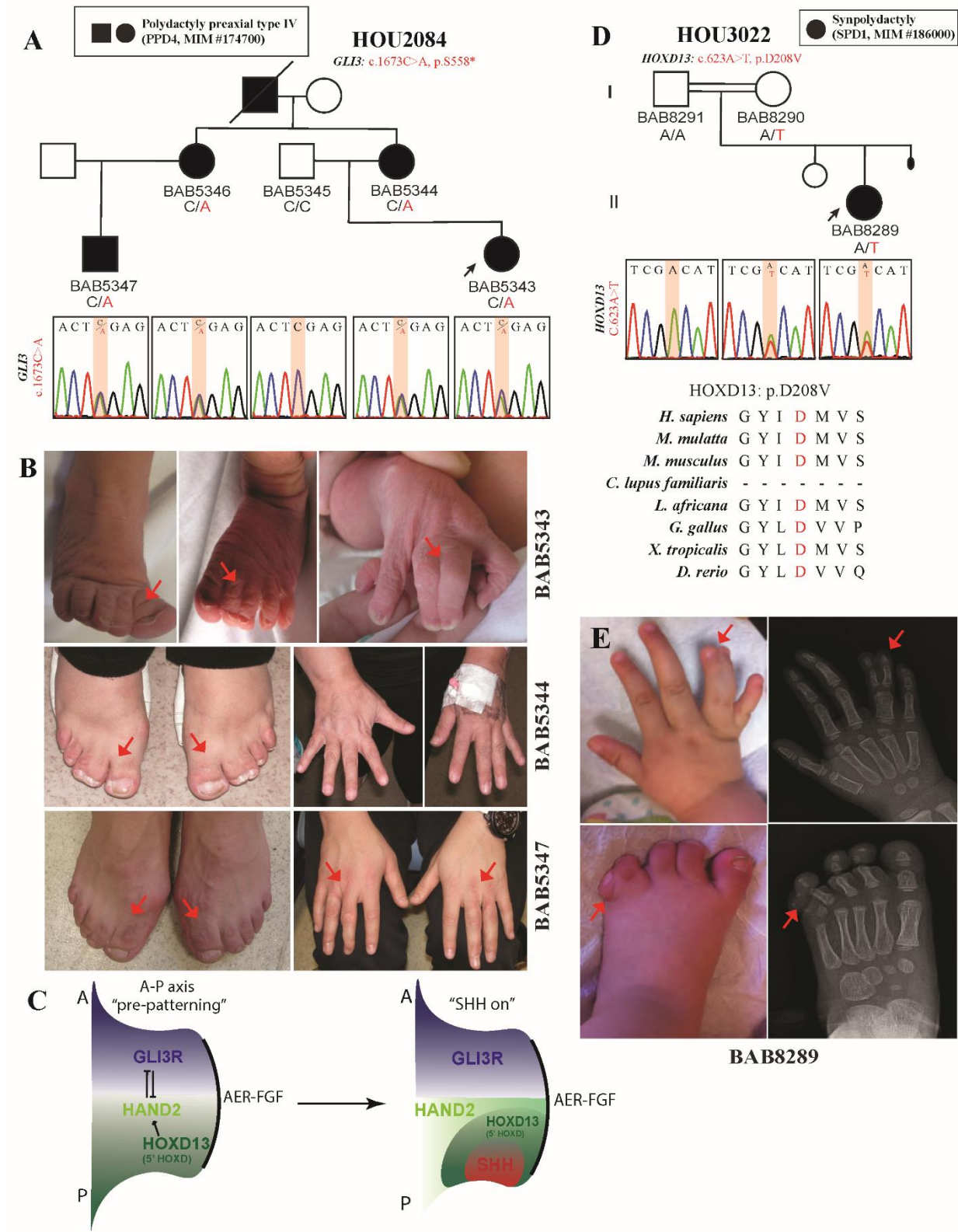

Figure S1. Novel variant identification involving genes contributing to A/P axis patterning

- A. A simplified pedigree of HOU2084 with selected family members. Novel nonsense variant (*GLI3*, c.1673C>A) profiled by four-color Sanger sequencing chromatogram, segregated with the PPD4 (MIM#174700) rare disease trait in accordance with Mendelian expectations for a dominant trait.
- B. Clinical photos show the dorsal views of hands and feet from three selected affected individuals. From top to bottom: proband (BAB5343), affected mother of the proband (BAB5344), and maternal cousin of the proband (BAB5347). Red arrows identify the common congenital limb malformation (CLM) features shared by the three affected individuals, including broad/duplicated halluces and 2-3 toe syndactyly and 3-4 finger syndactyly (note post-surgical scar in the hand of subject BAB5347)
- C. The left illustration describes the limb bud. Mutual genetic antagonism between the short isoform of *GLI3* (*GLI3R*) and *HAND2* is required to establish a “pre-patterning” of the A/P axis before forming the *SHH*-ZPA gradient. *HOXD13* expression is essential to sustain the *HAND2* activation. The right diagram shows that the counterregulatory interactions among *GLI3R*, *HAND2* and *HOXD13* continue to orchestrate limb bud A/P polarization after establishing the *SHH* gradient.
- D. Pedigree structure of HOU3022/BAB8289 and segregation shows the maternally inherited missense variant allele (*HOXD13*, c.623A>T). The altered residue (p.D208V) is evolutionarily conserved.
- E. Clinical photos and radiographs show the observed SPD1 phenotype of proband BAB8289. Photos show 4th finger polysyndactyly and 4-5 toe syndactyly; radiographs of the hand show duplication of the 4th finger phalanges and hypoplasia of the distal phalanx of the 5th finger; radiographs of the feet show a delta proximal 5th phalanx (longitudinally bracketed epiphysis) and hypoplastic distal phalanges of the great toe and 5th toe.

Figure S2

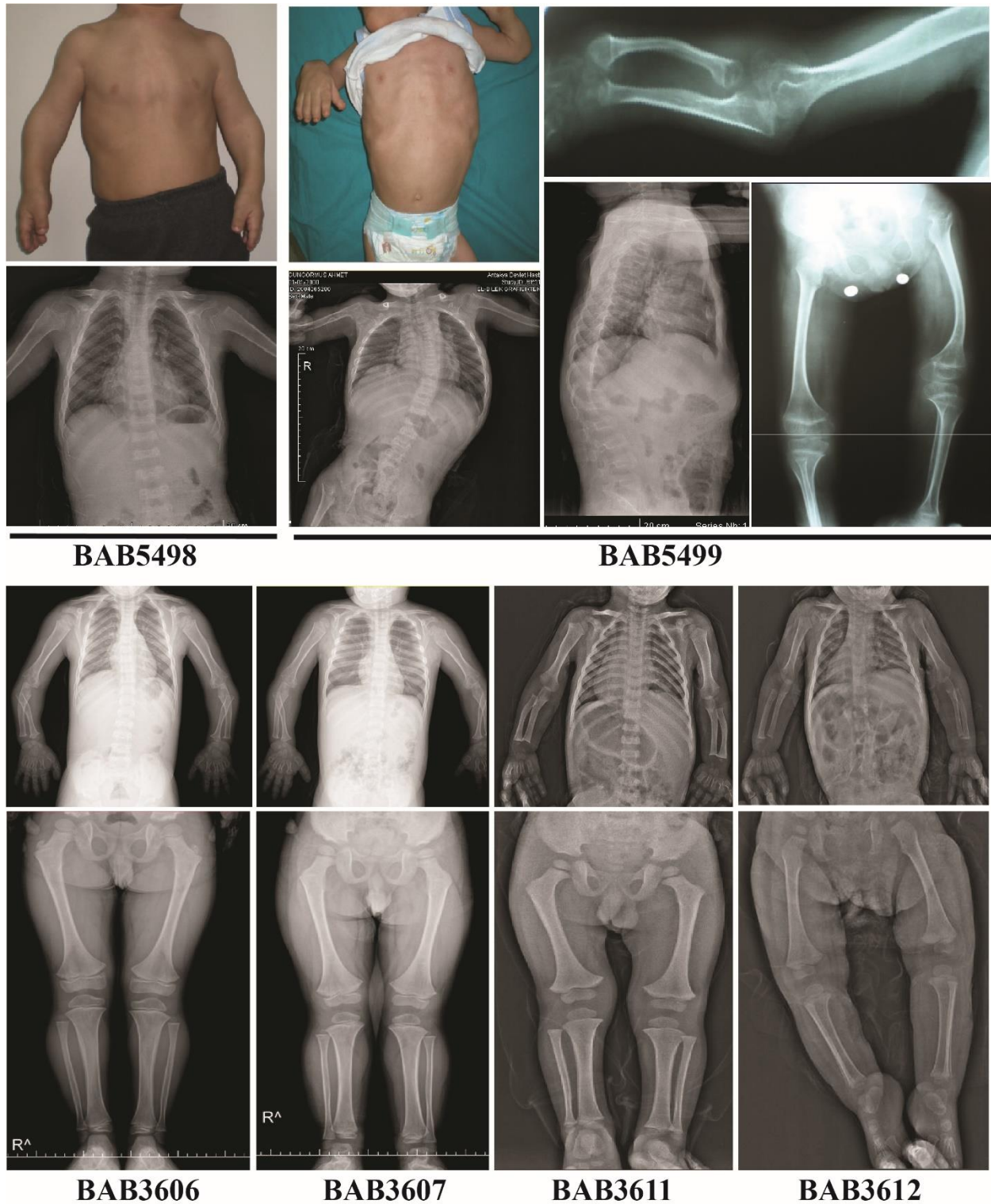

Figure S2. Clinical photographs and radiographs of six individuals with Acromesomelic dysplasia, Maroteaux type (AMDM) from three unrelated Turkish families. Upper panel:

BAB5498 and BAB5499 images demonstrate *pectus excavatum* and micromelia with predominant rhizomelia; radiographs show a short, bowed forearm, platyspondyly, thoracic kyphosis, and congenital hip dislocation in individual BAB5499. Bottom panel: Radiographs from four affected individuals show short upper limbs with short and thick humeri and even more pronounced shortening and bowing of the forearms, symmetrically short and broad metacarpals, and phalanges, varying degrees of platyspondyly, flattened acetabulum and *coxa vara* in individual BAB3612.

Figure S3

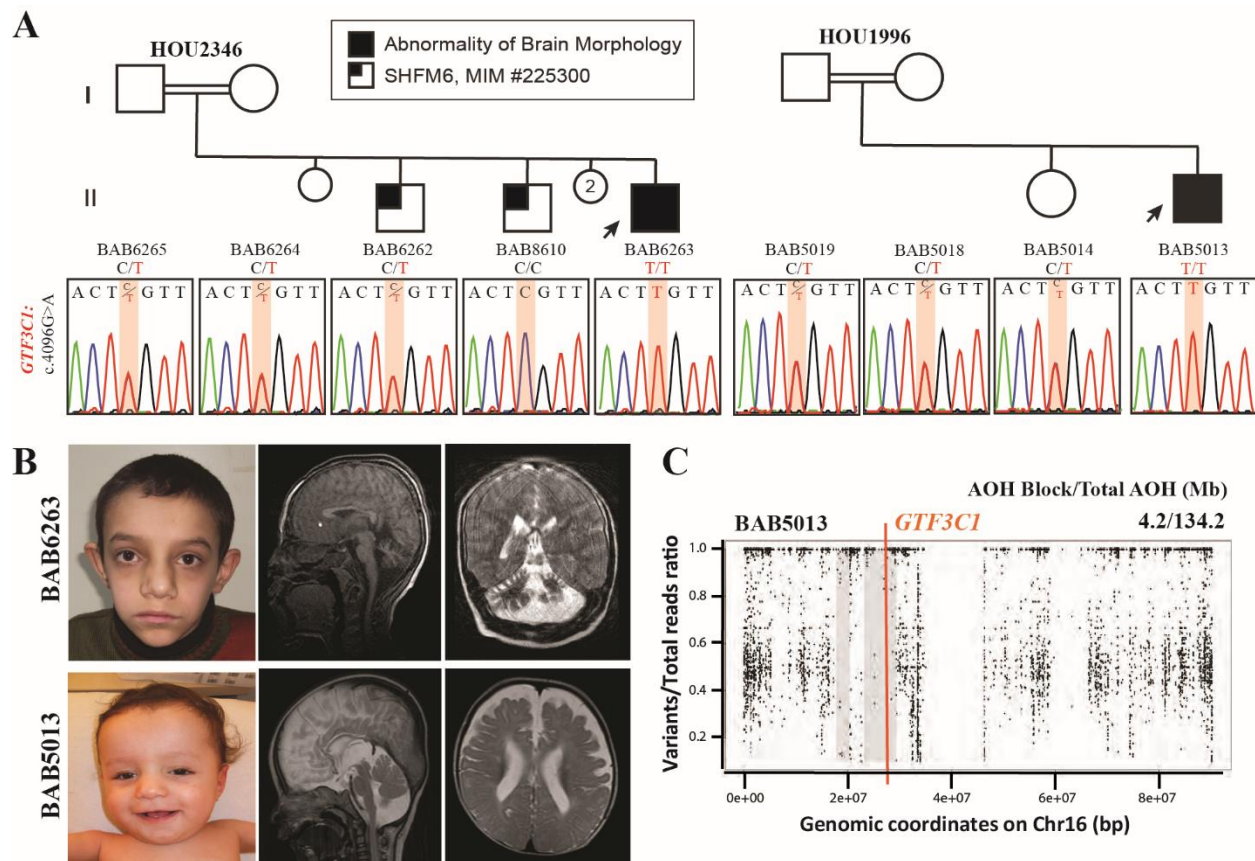

Figure S3. Pedigree, clinical photo, and segregation analysis of a homozygous missense variant of *GTF3C1* in two unrelated Turkish families. (A) Left: pedigree of HOU2346 with reported parental consanguinity. Missense variant (*GTF3C1*: c.4096G>A, p.E1366K) profiled by Sanger dideoxy sequencing, segregated with brain malformation in accordance with Mendelian

expectations for a recessive trait. Segregation was previously published by our group as the right pedigree of HOU1996 and the same variant (*GTF3C1*: c.4096G>A, p.E1366K). (B) The top panel shows the facial image of BAB6263, followed by brain MRIs demonstrating cerebellar atrophy. The bottom panel displays the facial appearance of BAB5013 and evidence of cerebral atrophy seen in the brain MRIs. (C) The AOH plot for BAB5013 demonstrates that the location of the missense variant allele of *GTF3C1* (marked by a red vertical line) is within a 4.2 Mb AOH block (denoted by the gray zone) on chromosome 16 with total AOH as 134.2 Mb.

A

Chr2: 171,524,396-171,524,412

BAB4814 WT/WT

HOU1397

BAB4813 WT/WT

BAB4812 WT/DEL

HOXD cluster

Genomic coordinates (Mb) (Chr2q)

B

(R) (L) (R) (L) (R) (L) (R) (L)

C

7.16 Mb de novo deletion

HOXD13 HOXD12 HOXD11 HOXD10 HOXD9 HOXD8 HOXD4 HOXD3 HOXD1

SP3 OLAI SP9 CIRI1 SCRN3 GPR155 WIPF1 CHRNA1 CHN1 ATEF ATP5B3 cTAD LVP EVX2 HOXD cluster MTX2 NFE2L2 HNRNP13 IGPS TTC30B TTC30A PDE11A

D

undelated allele 5' 3'

deleted allele

For Primer (jet) Rev. Primer (jet)

~1100 bp

~7.16 Mb

DNA Ladder

BAB4814 WT/WT

BAB4813 WT/WT

BAB4812 WT/DEL

Healthy gDNA control WT/WT

Negative control (No gDNA)

Control primer in unrelated region

E

chr2(+):171,524,379-171,524,412

AluSx (+) AGGCGGGTGGATCACCTAAGGTCAGGAGTTC

deleted allele AGGCGGGTGGATCACCTAATTTTAGGCATTC

For. Primer (jet) GCATAGA TTGTAAGAATAATTTTAGGCATTC

chr2(-):171,525,253-171,525,283

AluSc8 (-) GCCACCCTCTGGCTAATTTTTTTTTTTT

deleted allele TGTGAAC TATTAATAAGTTTTTTTTTTTTT

TGTGAAC TATTAATAAGTTTTTTTTTTTTT

3-bp microhomology

5-bp microhomology

Poly (T)*

F

Chr2:171524396-178694337

(Cen) AluSx (+) TS2 AluSc8 (-)

undelated allele

deleted allele

INV

TS1

~1100 bp

Figure S4. *De novo* heterozygous deletion mediated by AAMR encompassing the entire *HOXD* territory causing a syndromic SHFM5 phenotype.

- A. Pedigree structure of family HOU1397, and a customized HD-aCGH designed to interrogate Chr2q31.1 of the proband and parents, confirmed that this 7.16 Mb heterozygous deletion observed in the proband (area marked by green shade) was a *de novo* mutational event (both parents are ‘wild type’ normal).
- B. Clinical images and radiographs of proband BAB4812 show the bilateral split hands (missing 3rd and 4th digit rays).
- C. The illustration shows a magnified view of the genomic architecture and depicts the gene name/symbol, length, orientation, and position of each disrupted gene within this large deletion (Cen: centromere; Tel: telomere). The red rectangle highlights the linear structure of the *HOXD* gene cluster with two flanking triangles demarcating each side, shaping the two large adjacent gene deserts with a regulatory archipelago in which the regulatory TADs of the *HOXD* cluster were discovered.
- D. The diagram shows the positions of a flanking primer pair designed to capture the deleted allele by producing an amplicon size of ~1.1 kb encompassing the approximate breakpoint location estimated from aCGH data. A positive control primer pair was applied for PCR of an ~650 bp unrelated genomic region. Below the diagram is the 1% agarose electrophoresis of the PCR products. Only the proband DNA shows a specific band of ~1.1 kb, representing the deleted allele.
- E. Sanger sequencing from the ~1.1 kb PCR product of the proband revealed that the deleted allele recombinant junction encompasses three distinct sequences: two flanking *Alu* elements (*AluSx/AluSc8*) in the opposite orientation with an inverted sequence (~300bp downstream to the 5' *AluSx*+) inserted in the middle. The first breakpoint junction encompasses a 3-bp microhomology (three sequence above the Sanger sequencing trace from top to bottom denote the reference *AluSx*+ reference (colored in blue), the proband ‘chimeric’ sequence, and the inverted sequence (colored in brown). The second breakpoint junction (bottom), highlighting a 5-bp microhomeology (three sequence above the sequencing trace from top to bottom), denotes the *AluSc8*- reference sequence (colored in purple), the proband ‘chimeric’ sequence, and reference inverted sequence colored in brown.
- F. A schematic diagram elucidates the hypothesized formation of this DEL-INV-DEL structure

driven by an MMBIR-based, two-step template switching (TS) event mediated by two *Alu* elements in the opposite orientation.

Figure S5

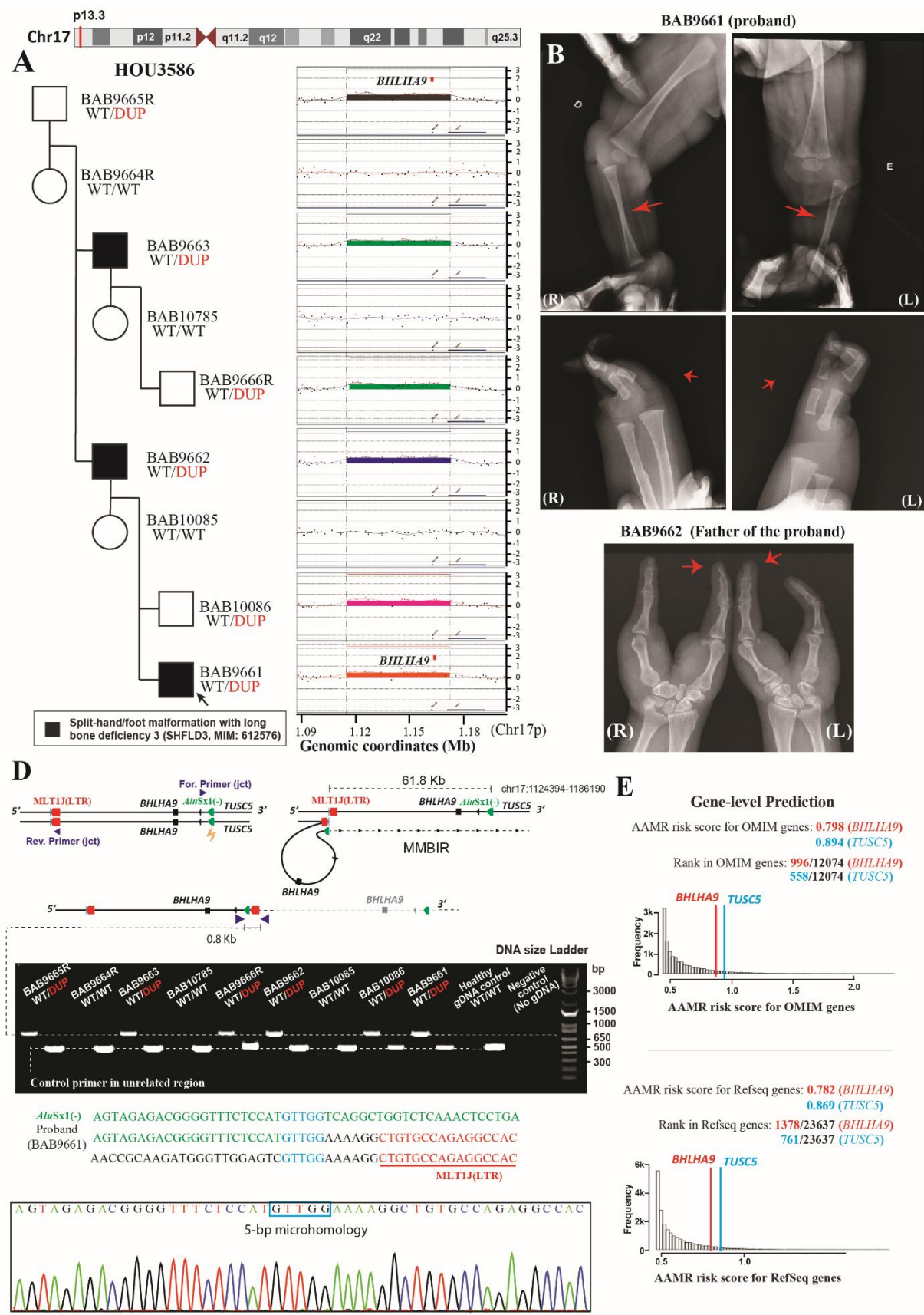

Figure S5: Heterozygous *BHLHA9* duplication in a Brazilian family causing SHFLD3 with reduced penetrance

- A. Pedigree structure of HOU3586 (F35 in da Rocha et al.³⁷) with an HD-aCGH interrogating Chr17p13.3 for each family member. A ~61.8 Kb duplication encompassing the full-length of *BHLHA9* and first exon of *TUSC5* was identified in six individuals following a paternal inheritance mode. However, only three of six duplication allele carriers (BAB9661(F35.1), BAB9662(F35.2) and BAB9663(F35.3)) manifest the SHFLD3 phenotype, consistent with the reduced penetrance frequently ascribed to this locus.
- B. Radiographs of lower and upper limbs of proband BAB9661(F35.1). The top two images demonstrate bilateral tibial hemimelia (marked by red arrows). The lower two images show the bilateral split hand and oligodactyly.
- C. Radiographs of the father BAB9662 (F35.2). There is bilateral split hand malformation with triphalangeal thumbs, oligodactyly and carpal bone abnormalities, including in the right-hand abnormal configuration of the trapezium and trapezoid, narrow capitate and broad and trapezoidal hamate, and the left-hand insertion of the capitate between the scaphoid and trapezoid bones and fusion of the hamate, triquetrum and pisiform bones.
- D. A schematic diagram shows the hypothetical model of *BHLHA9* tandem duplication formed by an MMBIR-based genomic rearrangement. A single-strand break, a one-ended double-stranded DNA molecule (oeDNA) likely generated from a collapsed fork, occurred in an *AluSx1*- element (yellow mark) and introduced a microhomology (marked by blue bar)-mediated template switching into a 61.8 kb upstream region. A primer set was designed to capture the tandem duplicated allele by PCR, producing an amplicon size of ~0.8 kb encompassing the approximate breakpoint location. Control primer pair was applied on a ~500 bp unrelated genomic region. Below the diagram is the 1% agarose electrophoresis of the PCR product; the specific band of ~800bp is the tandem duplicated allele captured only in six duplication carriers. Sanger sequencing (bottom) from the ~800bp PCR product of the proband revealed a 5-bp microhomology. Three sequences above the Sanger sequencing trace from top to bottom denote the 3' *AluSx1*+ reference (colored in green), the proband 'chimeric' sequence, and the 5' reference sequence (adjacent downstream LTR colored in red).
- E. Gene-level prediction from AluAluCNVPredictor shows the machine learning elevated

AAMR relative risk score of *BHLHA9* and *TUSC5* in OMIM genes (0.798 and 0.894) and in RefSeq genes (0.782 and 0.869), respectively. This score represents a human genome genomic instability risk.

Figure S6

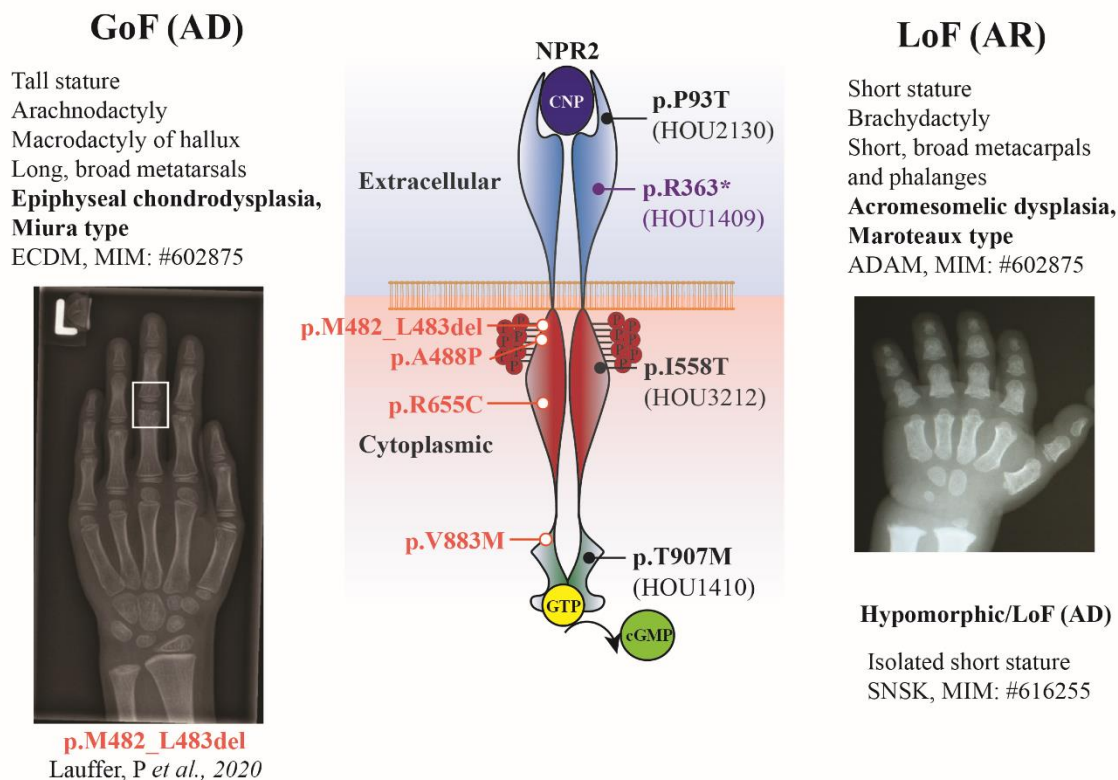

Figure S6. An illustration shows the “mirror trait” produced by the allelic series of *NPR2*. This diagram shows a typical model for a ‘mirror trait’ denoted as the opposite extremes of the phenotype observed in association with LoF versus GoF alleles. Biallelic LoF variants of *NPR2* reported in this study result in AMDM primarily due to dysregulation of CNP-NPR2-cGMP signaling homeostasis. The patient affected by AMDM shows short stature and acromesomelia featured by short and broad metacarpals and phalanges. However, the mirror opposite disease trait was observed in the patient who carried a heterozygous GoF variant and manifested tall stature, arachnodactyly, extra epiphyses, and elongated metacarpals and phalanges due to elevated hormonal catalytic activity.

Figure S7

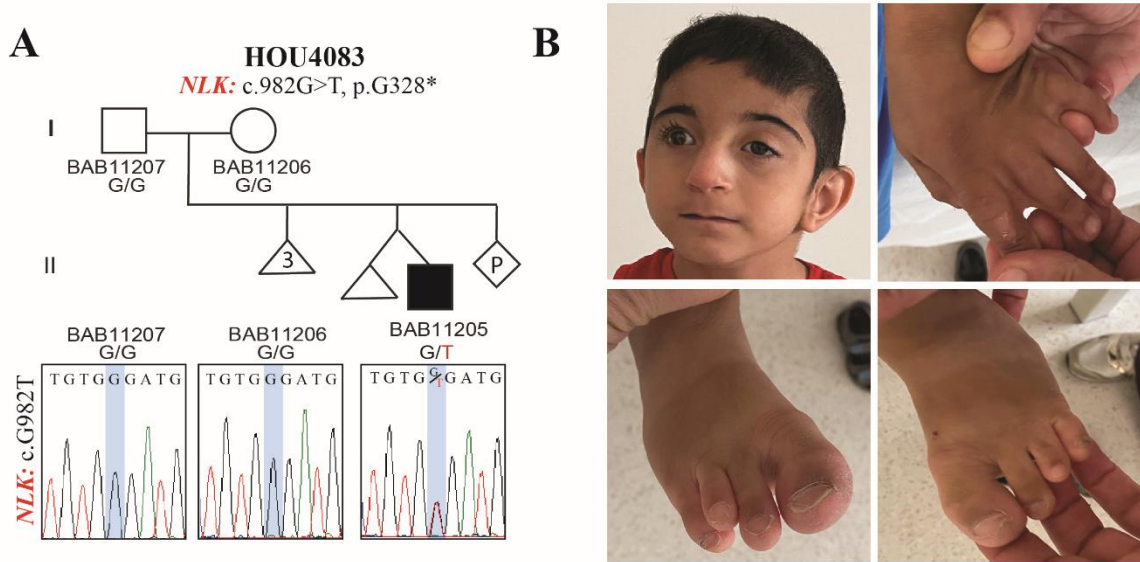

Figure S7. Pedigree, clinical photo, and segregation analysis of a *de novo* nonsense variant of *NLK* in a Turkish family. (A) Pedigree of HOU4083 and variant information was reported from our group. Below the pedigree is the Sanger sequencing confirmation showing the *de novo* nonsense variant (*NLK*: c.982G>T, p.G328*) is only present in the proband (BAB11205). (B) Clinical photos of BAB11205 manifest unilateral left microphthalmia, 2nd/3rd finger syndactyly on the left hand, and bilateral foot oligodactyly.

SUPPLEMENTAL MATERIAL AND METHODS

PCR Primers that were utilized for this study

Gene/Locus	Coordinate (hg19),<br>Nucleotide Change	Forward Primer	Reverse Primer
<i>GLI3</i>	Chr7:42017296_G>T	TTTCCAGTCCCACCTA<br>GGAA	AAAAGCTGCTGACC<br>CTTGAA
<i>GTF3C1</i>	Chr16:27492500_C>T	CTGAAGGACCAATGG<br>GAGAA	CAGGTACCCCAGTT<br>TCCTGA
<i>HOXD13</i>	Chr2:176958241_A>T	TACAGCAGAATGCGC<br>TCAAG	CACAACTCCCCTC<br>CCAAGT
Junction_Chr17p13.3<br>(HOU3358)	Chr17:1164471-<br>1239336; Duplication	GACTGCACCTCATCT<br>CACTAATCA	CATCCTCCATCATG<br>AACAAGAG
Junction_Chr17p13.3<br>(HOU3586)	Chr17:1124394-<br>1186190; Duplication	CTTTTCCCGTGCTCTT<br>TCTG	GTCTCCCAGGTTCA<br>TGCAAT
Junction_Chr2q31.1	Chr2:171524396-<br>178694337; Deletion	CCCCATTGCCTTGTTA<br>ATTG	TCACTCCAGTTGGC<br>TGTGAC
<i>NPR2</i>	Chr9:35802243_T>C	CCCACCATTTTCATCCT<br>GTCT	GCAAATGTTGGGAG<br>GGTCTA
<i>NPR2</i>	Chr9:35792682_C>A	GCACTAGCTGTGGAG<br>GCTCT	TGAAGTGCCCGTGT<br>AGTGTC
<i>NPR2</i>	Chr9:35800118_C>T	CATCAAGTCTTGGCT<br>GTGGA	GTGGGCAACTCTGC<br>CATATT
<i>NPR2</i>	Chr9:35808513_C>T	TCACCAGCCTCTGTC<br>CTCTT	CAGACTGGCCCTTT<br>GAGAAG
<i>WNT10B</i>	Chr12:49360306_TG>T	CAGCACATAGCAGCA<br>CCAGT	CAGCACATAGCAGC<br>ACCAGT
